# Supplementary material for: Flexible and digestible wood caused by viral-induced alteration of cell wall composition
Source: Curr Biol. 2022 Aug 8;32(15):3398–3406.e6. doi: 10.1016/j.cub.2022.06.005 (PMC9616729; doi:10.1016/j.cub.2022.06.005)
Supplement: Document S1. Figures S1–S3 and Tables S3 and S4 [file mmc1.pdf]

Current Biology, Volume *32*

Supplemental Information

Flexible and digestible wood caused by viral-induced alteration of cell wall composition

Holly Allen, Leo Zeef, Kris Morreel, Geert Goeminne, Manoj Kumar, Leonardo D. Gomez, Andrew P. Dean, Axel Eckmann, Cinzia Casiraghi, Simon J. McQueen-Mason, Wout Boerjan, and Simon R. Turner

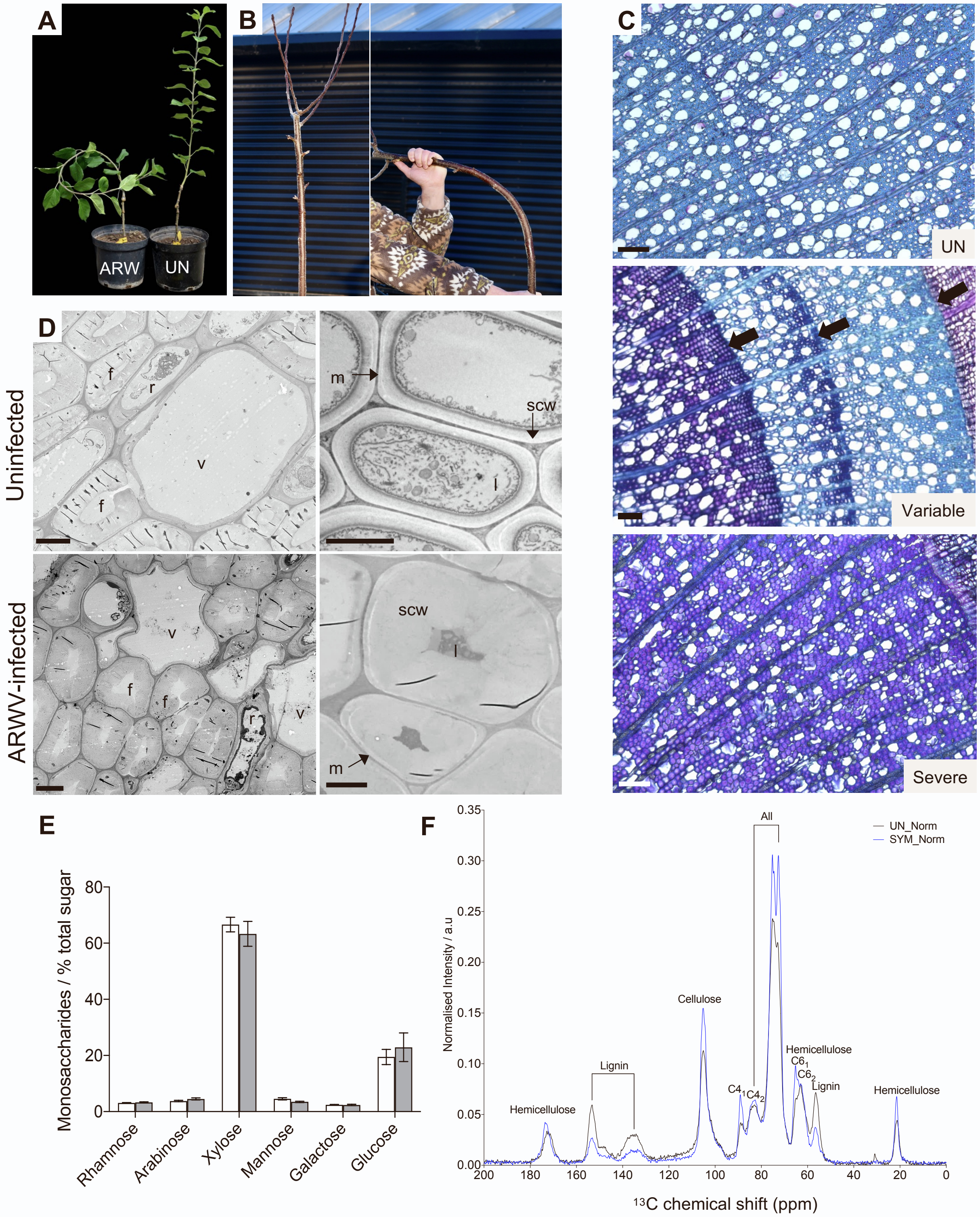

Figure S1 Symptom distribution in ARWV-infected branches and cell wall composition. Related to Figure 1. (**A**) Uninfected (UN) and ARW-infected (ARW) apple tree grafts. The ARW graft has been bent to demonstrate the stem flexibility. **(B)** 4 year old symptomatic branch from ARW-infected apple tree grafts growing unaided (left hand side) and bent to demonstrate the stem flexibility (right hand side) **(C)** Representative apple xylem cross-sections stained with 0.05% Toluidine blue taken from uninfected branches and ARWV-infected branches exhibiting variable or severe symptoms. Asymptomatic regions of infected material resemble the uninfected, while cell walls of symptomatic fibres stain a purple colour. Arrow indicates the growth ring. While year 1 growth exhibits clear symptoms, symptoms are only apparent at the end of year 2 and absent from year 3. Scale bars = 50 µm **(D)** Transmission electron microscopy of apple xylem. Scale bars = 5 µm (left panel) and 3 µm (right panel). Fibres (f), ray cells (r), vessels (v), secondary cell wall (scw), mid lamellae (m) and cell lumen (l) are indicated. **(E)** Monosaccharide composition of xylem from uninfected (white bars) and symptomatic xylem (grey bars). **(F)** Normalized spectra of solid-state ^{13}C NMR analysis from uninfected (black) and symptomatic (blue) cell walls.

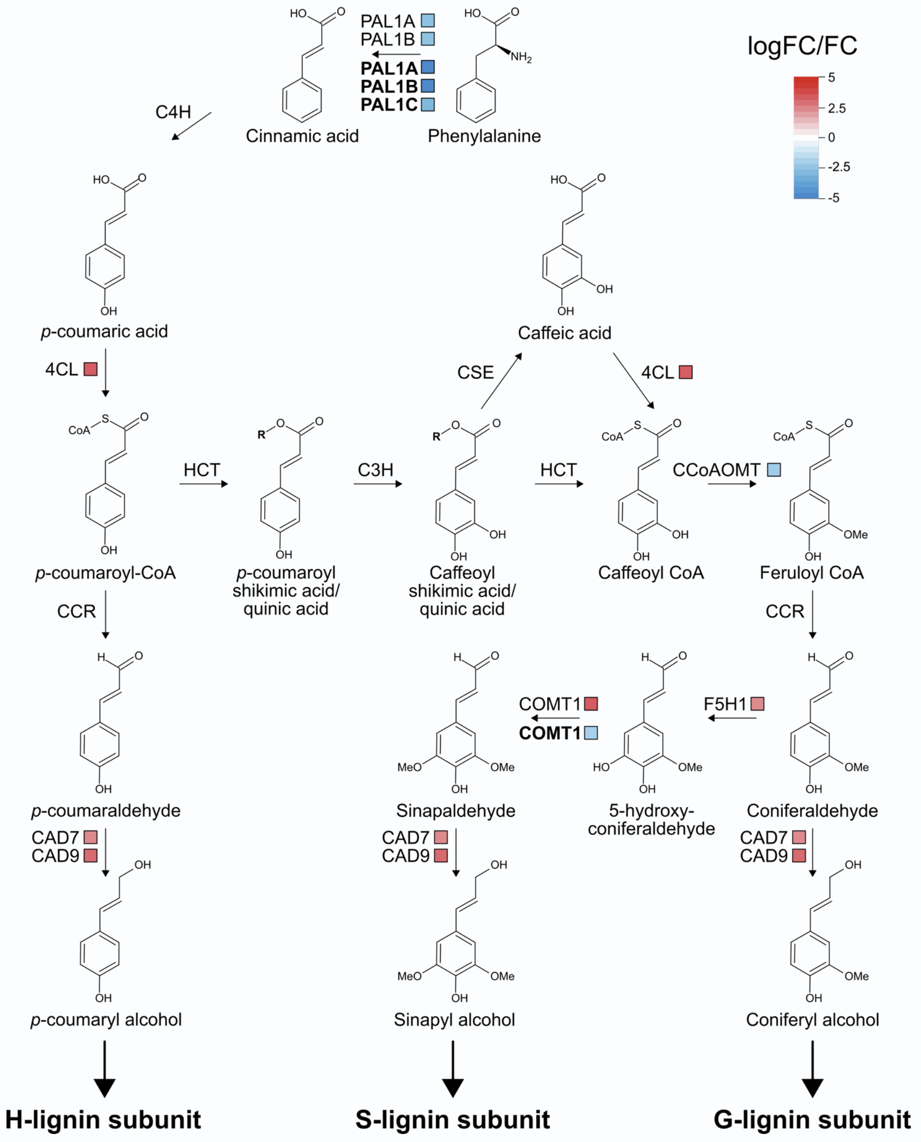

Figure S2 Expression of lignin biosynthetic genes and proteins in apple xylem. Related to Table 1. Each square represents the expression of a different gene (regular font) or protein (bold). Log fold change (FC) in gene expression and FC in protein abundance is expressed in terms of symptomatic xylem relative to uninfected xylem. All genes exhibit significantly altered expression with an FDR-adjusted p-value ≤ 0.01 and logFC ± 2. All proteins exhibit significantly altered abundance with a p-value ≤ 0.05 and FC ± 2.

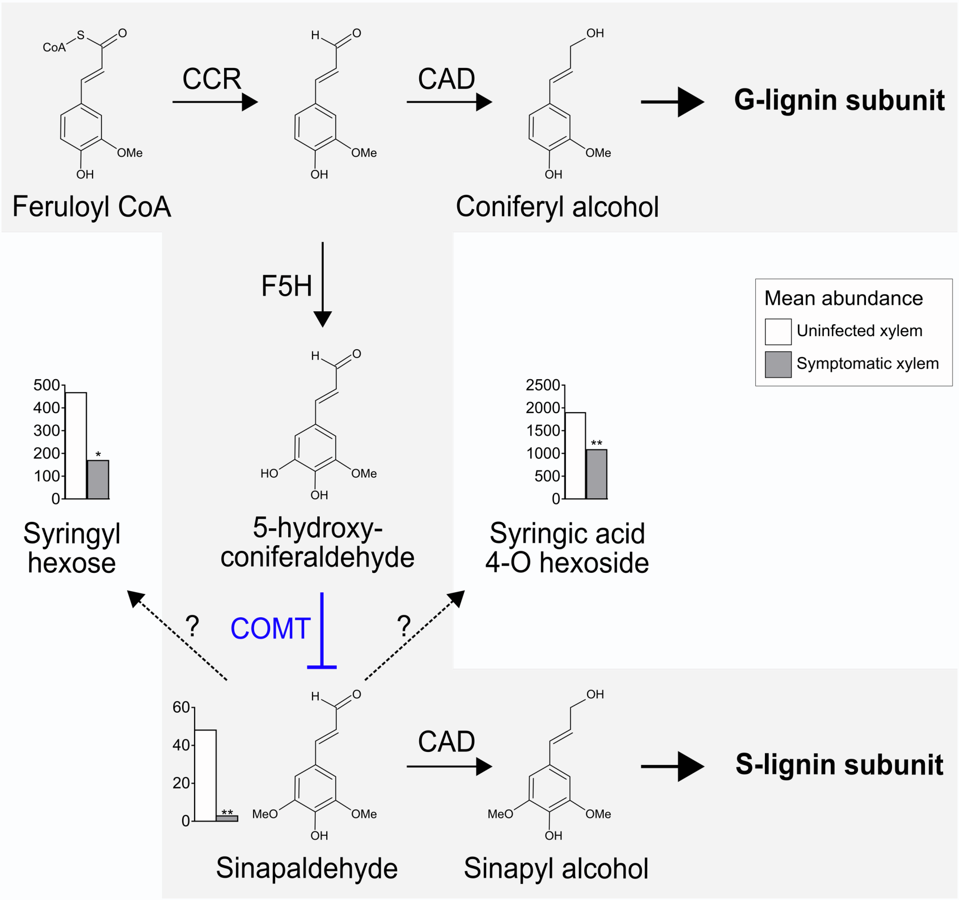

Metabolite ID	Putative name	Type	FC	Mean abundance	
				UN	SYM
2.74_359.0981m/z	syringic acid 4-O-hexoside	P	0.57	1907.56	1093.77
3.52_359.0980m/z	syringoyl hexose	P	0.37	469.20	171.49
9.99_207.0666m/z	sinapaldehyde	P	0.06	48.06	2.83

Figure S3 Disruption in the late stages of lignin biosynthesis in symptomatic xylem. Related to Figure 2. Arrows represent enzymatic steps during the late stages of lignin biosynthesis. Dashed arrows indicate steps that are uncharacterised. Graphs show mean metabolites abundance.. Asterisks indicate significance based on a T-test (*, p ≤ 0.05, **, p ≤ 0.01). Table shows metabolites that have a fold-change with a p-value < 0.05. Abbreviations: CCR = cinnamoyl-CoA reductase, CAD = cinnamyl alcohol dehydrogenase, FSH = ferulate 5-hydroxylase and COMT = caffeic acid 3-O-methyltransferase.

Metabolite ID	Putative name	Type	FC	Mean abundance	
				UN	SYM
8.76_687.1558m/z	-	U	17.65	9.12	160.94
8.56_223.0615m/z	2[hydroxy(phenyl)methyl] butanedioic acid	P	11.77	164.88	1941.25
5.12_239.0561m/z	2-[hydroxy(hydroxyphenyl)methyl] butanedioic acid	P	6.62	47.53	314.76
6.48_385.1140m/z	phenylacetyl-hexuronosyl-glycerol	P	5.82	59.07	343.81
7.74_385.1141m/z	phenylacetyl-hexuronosyl-glycerol	P	5.67	365.89	2073.74
15.86_461.1090m/z	-	U	3.91	41.41	161.91
6.36_251.0562m/z	2-oxo-3[hydroxy(phenyl)methyl] pentanedioic acid	P	3.74	137.30	513.18
4.01_465.1032m/z	taxifolin hexoside	F	3.29	120.68	396.67
12.05_883.2292m/z	-	U	2.40	63.23	151.53
1.05_551.1810m/z	-	U	2.39	126.68	302.42
13.94_301.0356m/z	quercetin	F	2.30	44.50	102.37
2.87_359.0617m/z	-	U	2.21	216.96	479.60
3.95_327.1084m/z	dihydro-*p*-coumaric acid hexoside	P	2.20	47.37	103.99
12.05_447.0938m/z	quercetin-O-methyl ether pentoside	F	2.07	156.84	324.62
9.60_793.1869m/z	-	U	2.06	68.22	140.62
11.46_723.1923m/z	-	U	2.04	85.27	173.58
12.97_741.2603m/z	-	U	0.49	238.55	116.02
10.93_615.1853m/z	-	U	0.44	154.62	67.70
7.27_307.1400m/z	-	U	0.41	166.20	67.82
18.44_839.3107m/z	-	U	0.36	193.79	70.44
5.90_419.1559m/z	-	U	0.35	299.06	105.83
16.57_855.3037m/z	tetralignol	O	0.34	230.42	78.22
18.71_809.3003m/z	**G(8-O-4)S(8-8)S(4-O-8)G**	O	0.31	781.92	240.37
16.17_855.3061m/z	**G(8-O-4)S(8-O-4)S(8-5)G**	O	0.29	189.08	55.67
7.64_613.2131m/z	**G(8-O-4)S hexoside**	O	0.27	585.15	157.10
8.24_567.2080m/z	**G(8red-5)G-hexoside**	O	0.25	135.03	34.07
15.69_825.2959m/z	**G(8-O-4)G(8-O-4)S(8-5)G**	O	0.24	366.13	86.07
16.32_825.2952m/z	tetralignol	O	0.22	240.22	52.84

Metabolite ID	Putative name	Type	FC	Mean abundance	
				UN	SYM
2.18_164.0718m/z	phenylalanine	P	18.48	15.71	290.31
7.41_206.0825m/z	N-acetylphenylalanine	P	3.61	20.03	72.39
2.74_359.0981m/z	syringic acid 4-O-hexoside	P	0.57	1907.56	1093.77
9.99_207.0666m/z	sinapaldehyde	P	0.06	48.06	2.83

Table S3 Differentially abundant phenolic metabolites in symptomatic xylem. Related to Figure 2. Fold change (FC) is expressed in terms of symptomatic xylem (SYM) relative to uninfected (UN) xylem. Compounds in bold are annotated with high confidence based on MS/MS spectral library identity matching, and compounds not in bold are putative structures based on MS/MS spectral similarity to library spectra and structural elucidation. The upper table shows metabolites that have a greater than two-fold change in abundance and a minimum abundance of 100 in either symptomatic or uninfected xylem with a p-value ≤ 0.001, while in the lower table the metabolites have a p-value < 0.01. Abbreviations: P = phenylpropanoid, F = flavonoids, O = oligolignols and U = unassigned.

sRNA sequence	ARW-1	ARW-2	ARW-3	ARW-4	Total
CTCTGACTGGTCTCACAACTG	1718	2008	710	170	4606
ATAGTCTACCTGCAGCATTTA	290	616	485	175	1566
CATCACCATCTTTCTAACAAA	260	473	243	89	1065
CCACCGGTTCTTGCATTCT	137	632	196	37	1002
ATAGTCTACCTGCAGCATTTAA	142	359	222	77	800
CTCATGGCTGAAGGAAATTTC	187	386	191	36	800
CTAGAGGGAACATTCGAGAGG	73	360	152	47	632
CCACCGGTTCTTGCATTCTAG	95	387	126	22	630
GCCACCGGTTCTTGCATTCTA	140	192	104	51	487
GCTGTTGGCCACCGGTTCTTG	58	234	141	52	485
CCTTAAGTCATGGAGTCAGCAA	180	168	117	11	476
CATCACAGCTGTTGGCCACCG	111	182	124	21	438
CTAGAGGGAACATTCGAGAGGA	39	273	92	26	430
TCATCACCATCTTTCTAACAAA	70	181	112	63	426
CCTGGACTCTAAGATCAACTT	92	235	50	19	396
ACGACTATAGAAAAACTGCATT	123	151	88	11	373
CCTTAAGTCATGGAGTCAGCA	107	124	91	28	350
GCCACCGGTTCTTGCATTCTAG	67	160	81	34	342
CACCGGTTCTTGCATTCTAGA	61	205	48	18	332
CTTTGCAGAAGAGTCCATTAG	96	155	49	12	312
CATCACCATCTTTCTAACAAAC	49	153	57	50	309
TCATCACCATCTTTCTAACAA	42	133	55	64	294
TCATGCCTAGAGGATCTGAAG	74	121	56	11	262
CAGCTGTTGGCCACCGGTTCT	26	95	85	18	224
TCACTCTGACTGGTCTCACAAC	36	112	46	11	205
TTTCTGCAACTAACTTCAAAGC	47	73	56	18	194
CTGTTGGCCACCGGTTCTTGC	26	85	62	18	191
TAGAGGGAACATTCGAGAGGA	14	110	33	13	170
TCAAGATAAGTAGGCAGCCTTA	31	82	42	11	166
TTGGCCACCGGTTCTTGCATT	22	70	49	25	166
TCACCATCTTTCTAACAAACA	43	67	15	28	153
GAAGTTAAGCCAGCAGATTTA	25	66	33	19	143
GGGAGATTCTGGAACGAGCTA	32	80	19	12	143
CTTAAGTCATGGAGTCAGCAA	27	70	30	14	141
TTCAGCACTGGGGATAATGCC	29	57	40	11	137
ATGCTTTCATCACCATCTTTC	21	45	46	23	135
CATCACCATCTTTCTAACAA	44	35	37	10	126
TTAATCAAGATAAGTAGGCAGC	17	61	28	10	116
GCAATTCAGGTAGTGCAGAGT	32	35	36	12	115
AGCTGTTGGCCACCGGTTCTTG	24	51	19	20	114
TAAGTCATGGAGTCAGCAA	19	61	20	12	112

sRNA sequence	ARW-1	ARW-2	ARW-3	ARW-4	Total
GCAATTCAGGTAGTGCAGAGTG	18	56	18	14	106
TCATCACCATCTTTCTAACA	23	32	19	26	100
TTCATCACCATCTTTCTAACAAA	13	45	18	12	88
TTCATCACCATCTTTCTAACAA	10	38	17	20	85
TTCATCACCATCTTTCTAACA	16	23	29	14	82
TCACCATCTTTCTAACAAAC	21	26	16	10	73
CTGTTGGCCACCGGTTCTTGCA	10	31	15	12	68
TTTCATCACCATCTTTCTAAC	11	15	13	16	55
TAATCAAGATAAGTAGGCAGC	11	21	10	10	52

Table S4 sRNAs mapping to the ARWV. Related to Table 2. All sRNA sequences were found in each ARWV-infected wood sample with more than 10 reads per samples. No reads were detected in UN samples.
