# Supplementary material for: Flexible and digestible wood caused by viral-induced alteration of cell wall composition
Source: Curr Biol. 2022 Aug 8;32(15):3398–3406.e6. doi: 10.1016/j.cub.2022.06.005 (PMC9616729; doi:10.1016/j.cub.2022.06.005)
Supplement: Document S2. Article plus supplemental information [file mmc5.pdf]

Current Biology

Report

Flexible and digestible wood caused by viral-induced alteration of cell wall composition

Highlights

- Apple rubbery wood (ARW) symptoms are caused by decreased lignin in woody tissue
- RNA-seq, proteomics, and metabolomics suggest phenylalanine levels decrease
- Virus-activated small interfering RNAs (vasiRNAs) are generated in response to ARWV infection
- VasiRNAs cause siRNA-based downregulation of phenylalanine ammonia

Authors

Holly Allen, Leo Zeef, Kris Morreel, ..., Simon J. McQueen-Mason, Wout Boerjan, Simon R. Turner

Correspondence

simon.turner@manchester.ac.uk

In brief

Apple rubbery wood (ARW) infections cause the branches to become abnormally flexible. Allen et al. demonstrate that the symptoms are caused by a specific decrease in lignin deposition, resulting from decreased flux through phenylalanine ammonia lyase (PAL). PAL is downregulated by siRNAs that are generated by the plant upon ARW infection.

Allen et al., 2022, Current Biology *32*, 3398–3406
August 8, 2022 © 2022 The Authors. Published by Elsevier Inc.
https://doi.org/10.1016/j.cub.2022.06.005

CellPress

CellPress OPEN ACCESS

Current Biology

Report

Flexible and digestible wood caused by viral-induced alteration of cell wall composition

Holly Allen,[1,7] Leo Zeef,[1] Kris Morreel,[2,3] Geert Goeminne,[2,4] Manoj Kumar,[1] Leonardo D. Gomez,[5] Andrew P. Dean,[1,8] Axel Eckmann,[6] Cinzia Casiraghi,[6] Simon J. McQueen-Mason,[5] Wout Boerjan,[2,3] and Simon R. Turner[1,9,10,*]
[1]School of Biological Science, University of Manchester, Oxford Road, Manchester M13 9PT, UK
[2]Department of Plant Biotechnology and Bioinformatics, Ghent University, Technologiepark 71, 9052 Ghent, Belgium
[3]VIB Center for Plant Systems Biology, Technologiepark 71, 9052 Ghent, Belgium
[4]VIB Metabolomics Core Gent, VIB, 9052 Zwijnaarde, Belgium
[5]Centre for Novel Agricultural Products (CNAP), Department of Biology, University of York, York YO10 5DD, UK
[6]Department of Chemistry, University of Manchester, Oxford Road, Manchester M13 9PT, UK
[7]Present address: Eberly College of Science, Pennsylvania State University, University Park, PA 16802, USA
[8]Present address: Department of Natural Sciences, Manchester Metropolitan University, Manchester M15 6BH, UK
[9]Twitter: @SRTurner_Dr_irx
[10]Lead contact
*Correspondence: simon.turner@manchester.ac.uk
https://doi.org/10.1016/j.cub.2022.06.005

SUMMARY

Woody plant material represents a vast renewable resource that has the potential to produce biofuels and other bio-based products with favorable net CO_2 emissions.[1,2] Its potential has been demonstrated in a recent study that generated novel structural materials from flexible moldable wood.[3] Apple rubbery wood (ARW) disease is the result of a viral infection that causes woody stems to exhibit increased flexibility.[4] Although ARW disease is associated with the presence of an RNA virus[5] known as apple rubbery wood virus (ARWV), how the unique symptoms develop is unknown. We demonstrate that the symptoms of ARWV infections arise from reduced lignification within the secondary cell wall of xylem fibers and result in increased wood digestibility. In contrast, the mid-lamellae region and xylem ray cells are largely unaffected by the infection. Gene expression and proteomic data from symptomatic xylem clearly show the downregulation of phenylalanine ammonia lyase (PAL), the enzyme catalyzing the first committed step in the phenylpropanoid pathway leading to lignin biosynthesis. A large increase in soluble phenolics in symptomatic xylem, including the lignin precursor phenylalanine, is also consistent with PAL downregulation. ARWV infection results in the accumulation of many host-derived virus-activated small interfering RNAs (vasiRNAs). *PAL*-derived vasiRNAs are among the most abundant vasiRNAs in symptomatic xylem and are likely the cause of reduced PAL activity. Apparently, the mechanism used by the virus to alter lignin exhibits similarities to the RNAi strategy used to alter lignin in genetically modified trees to generate comparable improvements in wood properties.[6–8]

RESULTS AND DISCUSSION

Secondary xylem morphology is perturbed by apple rubbery wood disease

Concerns over the rising carbon footprint and depletion of nonrenewable fossil fuels have encouraged the use of cleaner and sustainable alternatives. Wood from trees represents a sustainable natural resource that has the potential to lock up carbon from the atmosphere and act as a feedstock for the synthesis of fuels and other novel chemicals and materials.[2,3,9] Although the woody secondary cell wall contains a high proportion of carbohydrates, the presence of the phenolic polymer lignin makes the cell wall recalcitrant to breakdown.[10] Consequently, chemical treatments are used prior to cellulose hydrolysis, increasing the expense and carbon footprint. Lignin biosynthesis has also been altered genetically,[6,11,12] and labile bonds have been introduced to make lignin more susceptible to breakdown;[8] however, these strategies rely on genetic modification and must overcome both public hostility and regulatory hurdles that have prevented the growth of genetically modified trees in many parts of the world.[13,14]

Apple trees infected with apple rubbery wood (ARW) disease exhibit abnormally flexible branches (Figures S1A and S1B). The disease is associated with the presence of apple rubbery wood virus (ARWV), a negative-stranded RNA virus belonging to the *Bunyaviridae* family.[5] Although ARW disease was largely eliminated from commercial rootstocks and it is no longer economically relevant, it remains of interest because of the highly unusual symptoms exhibited by infected trees. We are not aware of any other virus causing symptoms that are remotely similar to those exhibited by ARWV-infected trees.

Histochemical analysis of sections from uninfected branches shows the blue staining of cellulose by astra blue is masked by the red staining of lignin by basic fuchsins (Figure 1A); however,

 © 2022 The Authors. Published by Elsevier Inc.
This is an open access article under the CC BY license (http://creativecommons.org/licenses/by/4.0/).

CellPress
OPEN ACCESS

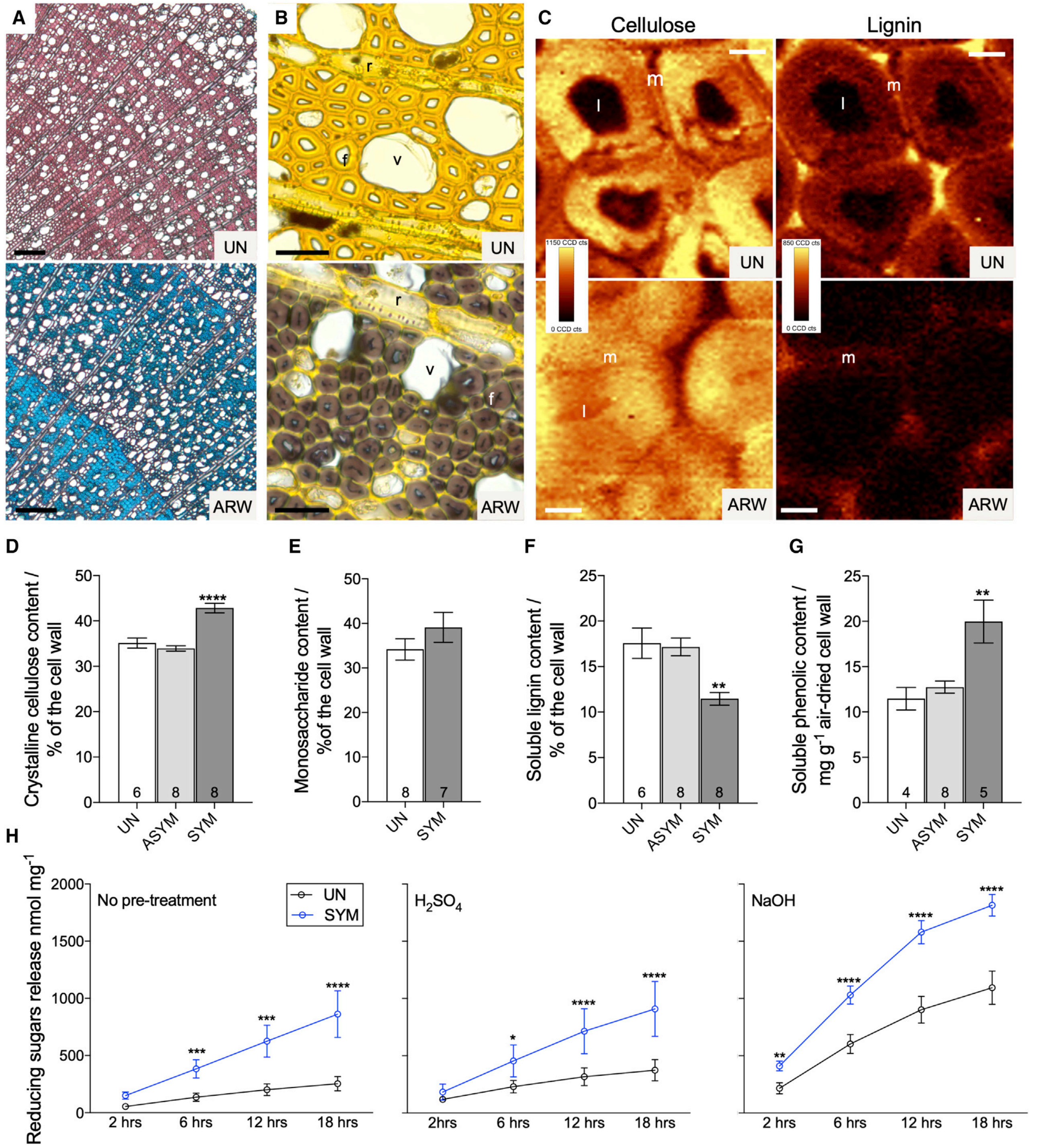

Figure 1. Symptoms of ARWV infection

(A–C) Histochemical staining (A and B) and Raman maps (C) of uninfected (top) and ARWV symptomatic xylem (bottom). Stains are astra blue/basic fuchsins that stains cellulose blue and lignin red (A) and $ZnCl_2$/KI that stains lignified cell walls yellow and cellulose purple. (C) Raman maps showing the distribution of cellulose and lignin, obtained by integrating the Raman signal between 2,770 and 3,040 cm^{-1} and 1,540 and 1,760 cm^{-1}, respectively. Scale bars indicate 100 μm (A), 25 μm (B), and 3 μm (C). Fibers (f), ray cells (r), vessel (v), cell lumen (l), and mid lamellae (m) are indicated.

(D–G) Cell wall composition of xylem from uninfected (UN), asymptomatic (ASYM), and symptomatic (SYM) ARWV-infected trees. Numbers on individual bars indicate the sample size.

(H) Sugar release from uninfected and symptomatic xylem with either no pre-treatment or pre-treatment with either acid (H_2SO_4) or alkali (NaOH).

(legend continued on next page)

sections from symptomatic pliable branches exhibit strong blue staining suggestive of decreased lignification. In symptomatic xylem, the poorly lignified fibers exhibit secondary cell walls that are much thicker than equivalent fibers from uninfected xylem or from adjacent vessels and ray cells, while the ray cells and mid lamellae of the fiber cells appear unaffected (Figure 1B). Closer examination with transmission electron microscopy revealed that the fiber secondary cell walls are so thick that in mature fibers almost no cell lumen remains (Figure S1D). This phenotype has been previously reported for other plants with altered lignin biosynthesis[15,16] and results from reduced lignification allowing other components to expand. Vessels also appear somewhat misshapen in symptomatic xylem (Figures 1B and S1D), though they retain an opening that is presumably sufficient for water transport. Furthermore, the thickness of the vessel cell wall is comparable with vessels in uninfected xylem (Figure S1D), suggesting vessel cell walls are largely unaffected. To confirm the histochemical analysis, Raman imaging was used to obtain a high-resolution measure of the distribution of cellulose and lignin. Although lignin is present throughout the cell walls of uninfected xylem, it is largely absent from the secondary cell walls in symptomatic xylem and is limited to the mid lamellae (Figure 1C).

Secondary cell walls in symptomatic xylem exhibit reduced lignin content

In many cases, the presence of symptoms was patchy along the length of the infected branch, and even in the most flexible branches, areas of asymptomatic xylem were still found (Figure S1C). To obtain the most accurate measure of cell wall alterations in symptomatic xylem, symptomatic and asymptomatic regions of the xylem were separated by dissection prior to biochemical analysis. Cell walls from symptomatic xylem had a 22%–26% higher crystalline cellulose content than either uninfected or asymptomatic xylem (Figure 1D). Total non-cellulosic monosaccharide content was marginally increased in symptomatic cell walls (Figure 1E), though the composition was unaltered (Figure S1E). Xylose content, which presumably derives from xylan, the major sugar of woody cell walls in most hardwoods,[17] was similar in uninfected and symptomatic xylem. Acetyl bromide-soluble lignin was reduced by a third in cell walls from symptomatic xylem (Figure 1F). Although this reduction in lignin is larger than previously reported, this likely reflects our use of only symptomatic xylem tissue.[18] Furthermore, the reduction of lignin specifically in the secondary cell walls of the xylem fibers is likely even greater than the 30% reduction in the total xylem. Although lignin content is substantially reduced, the soluble phenolic content of symptomatic xylem was approximately 2-fold higher than in non-symptomatic wood (Figure 1G). A specific decrease in lignin was confirmed by ^{13}C solid-state NMR (Figure S1F). Decreases in the heights of peaks with signals at 153, 133, and 56 ppm in the symptomatic spectra demonstrate that syringyl subunits connected by β-O-4 linkages and methoxyl groups of lignin monomers were reduced in symptomatic cell walls (Figure S1F). The reduction of lignin caused a proportional increase in the spectra for the remaining cell wall polysaccharides: cellulose and hemicellulose (Figures 1D and 1E).

Altered lignin can improve the yield of sugar released from cellulose hydrolysis.[6,19] Consequently, we assessed the digestibility of symptomatic xylem by measuring reducing sugars released by cell-wall-degrading enzymes. After 18 h, symptomatic xylem yielded 3-fold more sugar than uninfected xylem (Figure 1H). Pre-treatment with alkaline, but not acid, further enhanced sugar release. The quantity of sugar released from untreated symptomatic xylem was similar to that released from NaOH-treated uninfected xylem (Figure 1H). Reduced lignification of symptomatic xylem fibers was most likely the predominant factor that enhanced cellulose.[19]

PAL gene expression and protein abundances are reduced in symptomatic xylem

We performed RNA-sequencing analysis on RNA from newly differentiating symptomatic and uninfected xylem. Using a false discovery rate (FDR) adjusted p value < 0.01 and a log-fold change, ±2, 668 genes were differentially expressed (Table S1). Various genes involved in lignin biosynthesis were differentially expressed in symptomatic xylem. There are 6 predicted *PAL1* genes in apple; however, *PAL1A* and *PAL1B* are the most abundant in xylem and both are significantly downregulated in symptomatic xylem (Tables 1 and S1). In contrast, homologs *of 4CL, COMT1, F5H, CAD*, and *CHORISMATE MUTASE* 2 were upregulated in symptomatic xylem (Tables 1 and S1).

Proteomic analysis using the same material used for RNA-sequencing indicated that 111 proteins exhibited a greater than 2-fold change in abundance with a p value ≤ 0.05 (Table S2). 8 of these 111 proteins were associated with phenylpropanoid metabolism (Table 1). PAL1A and PAL1B exhibited a reduction in protein levels in symptomatic xylem of 13 and 11 fold, respectively,(Table S2), a slightly larger reduction than that seen for gene expression (Table 1). Strong reduction of PAL1 indicates that the first committed step in lignin biosynthesis may be compromised during ARWV infection. Peptides corresponding to caffeic acid 3-O-methyltransferase (COMT), another enzyme involved in lignin biosynthesis, were reduced by 2-fold in symptomatic xylem even though *COMT1* gene expression was over 8-fold higher (Table 1) and is the likely cause of reduced syringyl lignin in symptomatic xylem (Figure S3). Homologs of LAC15,[20] PRX25,[21] and PRX52[22] that are involved in polymerizing monolignol subunits in *Arabidopsis* were significantly more abundant in symptomatic xylem (Table 1).

Alteration in phenylpropanoids in symptomatic xylem is consistent with PAL downregulation

Of the approximately 2,774 profiled compound ions detected, 28 had a 2-fold change in abundance with a p value ≤ 0.001 and an average abundance of at least 100 in either uninfected or symptomatic xylem (Figure 2; Table S3). Several oligolignols

Comparisons were made using a one-way ANOVA and Tukey's multiple comparison test (D–G) or a two-way ANOVA and a Sidak's multiple comparison test (H). Asterisks indicate significance (p > 0.05, *p ≤ 0.05, **p ≤ 0.01, ***p ≤ 0.001, ****p ≤ 0.0001). Bars show standard error of the mean (D–G) or standard deviation (H).
See also Figure S1.

CellPress
OPEN ACCESS

Table 1. Expression of phenylpropanoid genes and proteins in apple xylem

Gene ID	Annotation	FC in gene expression	FC in protein abundance
Shikimate pathway			
MD08G1057400	chorismate mutase 2 (CM2)	51.00	N/A
Lignin biosynthetic pathway			
MD01G1106900	phenylalanine ammonia lyase 1A (PAL1A)	−5.12	−12.97
MD07G1172700	phenylalanine ammonia lyase 1B (PAL1B)	−4.95	−11.04
MD12G1116700	phenylalanine ammonia lyase 1C (PAL1C)	N/A	−3.18
MD13G1113700	4-coumarate-CoA ligase (4CL)	8.43	N/A
MD10G1064200	ferulate 5-hydroxylase 1 (F5H1)	4.62	N/A
MD07G1300200*	caffeic acid 3-O-methyltransferase 1 (COMT1)	8.85	−2.02*
MD10G1155000	cinnamyl alcohol dehydrogenase 7 (CAD7)	4.71	N/A
MD15G1008100	cinnamyl alcohol dehydrogenase 9 (CAD9)	7.02	N/A
MD01G1234900*	laccase 15 (LAC15)	N/A	5.31*
MD07G1230500	peroxidase 52 (PRX52)	N/A	4.03
MD15G1172800	peroxidase 25 (PRX25)	N/A	2.54
Flavonoid pathway			
MD06G1201700*	flavonoid 3′ mono-oxygenase	N/A	3.11*
MD03G1010700	dirigent protein 22	5.28	4.77

Fold change (FC) in transcript and protein abundance is expressed in terms of symptomatic xylem relative to uninfected xylem. All genes exhibit significantly altered expression, with an FDR-adjusted p value ≤ 0.01 and logFC ±2. All proteins exhibit significantly altered abundance, with a p value ≤ 0.05 and FC ±2. Genes and proteins not differentially expressed are indicated by N/A. Values marked with an * have multiple gene IDs because individual proteins could not be distinguished during the proteomic analysis: COMT1 (MD07G1300200, MD01G1229100, MD01G1219000, MD01G1229000, MD07G1209500, MD07G1209700, MD07G1300300, MD07G1301100), LAC15 (MD01G1234900, MD07G1307400, MD07G1308000), and flavonoid 3′-mono-oxygenase (MD06G1201700, MD14G1210700). See also Figure S2 and Tables S1 and S2.

were significantly reduced in symptomatic xylem to less than one-third of their abundance in uninfected xylem (Table S3). Decreased oligolignols and increased soluble phenolics have been observed previously in plants with altered lignification[23–25] and are consistent with the defect in symptomatic xylem resulting from a decrease in the flux of phenylpropanoids into lignin biosynthesis.

By lowering the significance threshold to $p \leq 0.01$, we found that the substrate for phenylalanine ammonia lyase (PAL), phenylalanine and *N*-acetylphenylalanine, a potential derivative of phenylalanine, were 18- and 3-fold more abundant in symptomatic xylem, respectively (Figure 2; Table S3), and they have been found to accumulate in *Arabidopsis* and *Petunia pal* mutants.[26,27] Two butanedioic acids exhibited the second- and third-largest fold increase in symptomatic xylem (Figure 2). Neither 2-[hydroxy(phenyl)methyl] butanedioic acid nor 2-[hydroxy(hydroxyphenyl)methyl] butanedioic acid has been previously reported in plants. Both phenylalanine and tyrosine are synthesized from chorismate, either in the chloroplast[28] or the cytoplasm[29] (Figure 2). We observed a 5-fold increase in the expression of *CHORISMATE MUTASE* 2 (Table 1), suggesting increased flux through the cytoplasmic pathway (Figure 2). The synthesis of phenylalanine via the cytoplasmic pathway occurs via phenylpyruvate (PPY) and hydroxyphenyl pyruvate (HPY) that differ only by the same hydroxyl group distinguishing the two butanedioic acids that accumulate in symptomatic xylem, suggesting that these novel compounds may be synthesized via PPY and HPP (Figure 2). The downregulation of PAL and the accumulation of phenylalanine and compounds derived from the shikimate pathway intermediates are all consistent with the suppression of PAL during ARWV infection (Figure 2).

Catechin is a well-characterized secondary metabolite that is relatively abundant in apple xylem and is increased by almost 2-fold in symptomatic xylem (Figure 2). Catechin synthesis requires coumaryl-CoA, a lignin biosynthesis intermediate generated downstream of PAL (Figure 2). The site of catechin biosynthesis in woody tissue is unknown, but it is unlikely to occur in fiber cells where phenylpropanoid flux is largely directed toward lignin biosynthesis for structural support. Ray cells are a

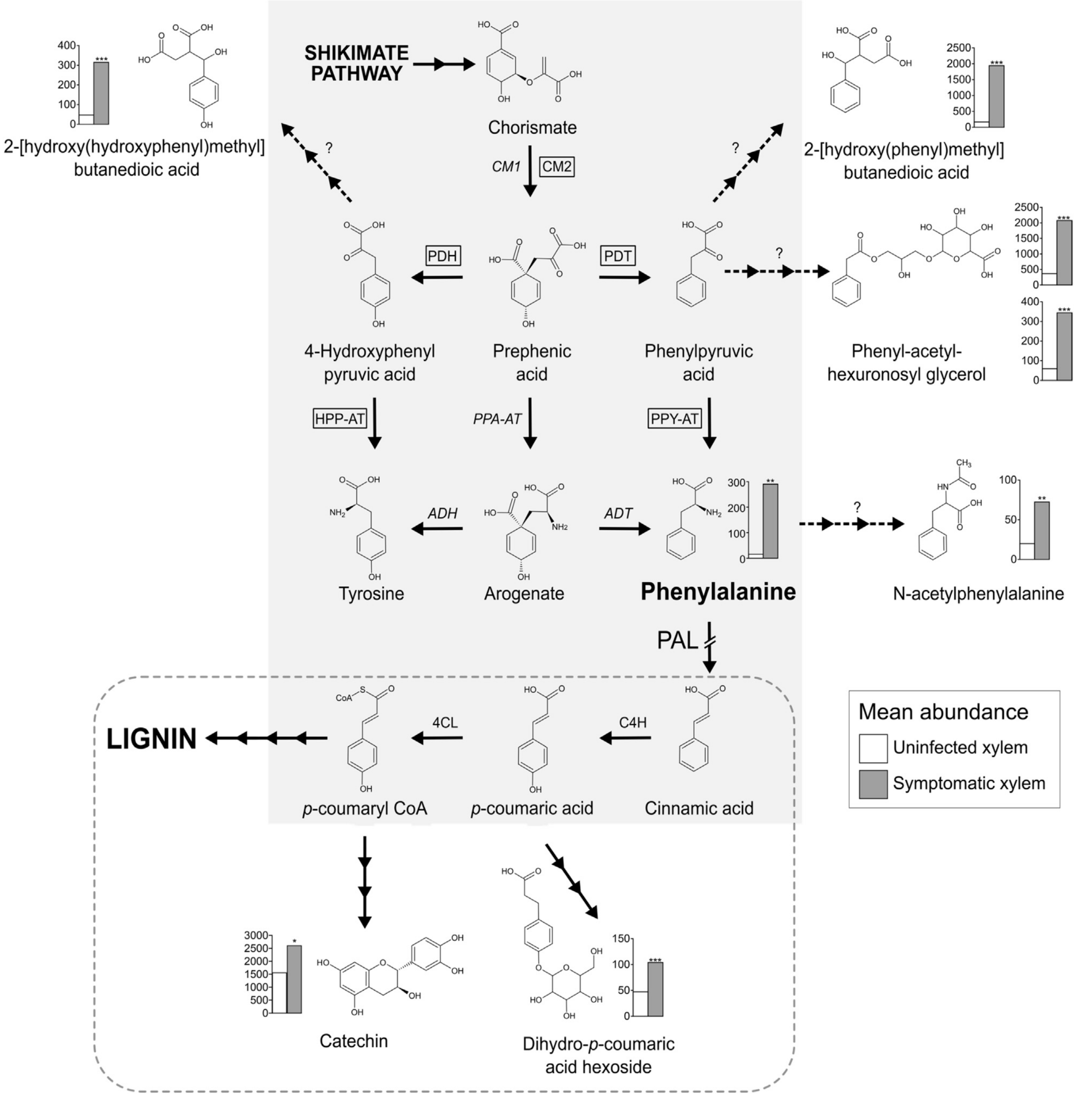

Figure 2. Potential disruption of phenylpropanoid biosynthesis in symptomatic xylem fibers

Arrows represent enzymatic steps in the shikimate and phenylpropanoid pathways (gray). Dashed arrows coupled with a question mark indicate steps that are uncharacterized in angiosperms. Enzyme names in boxes are involved in the chloroplastic pathway, while enzyme names in italics are involved in the cytosolic pathway for phenylalanine synthesis. In fibers exhibiting ARWV symptoms, the pathway to lignin biosynthesis is inhibited by downregulation of PAL. Those pathways proposed to take place in ray cells, which appear to be unaffected by ARWV, are enclosed by a dashed box. Graphs show mean abundance of metabolites in uninfected (white bars) and symptomatic (gray bars) xylem. Asterisks indicate significance based on a t test (*$p \leq 0.05$, **$p \leq 0.01$, ***$p \leq 0.001$). Abbreviations are as follows: CM, chorismite mutase; PDH, pyruvate dehydrogenase; PDT, prephenate dehydratase; HPP-AT, hydroxyphenylpyruvic acid aminotransferase; PPA-AT, prephenic acid aminotransferase; PPY-AT, phenylpyruvic acid aminotransferase; ADT, arogenate dehydratase; ADH, arogenate dehydrogenase; PAL, phenylalanine ammonia lyase; C4H, cinnamate-4-hydroxylase; and 4CL, 4-coumarate-CoA ligase.

See also Figure S3 and Table S3.

CellPress
OPEN ACCESS

Table 2. Changes in siRNAs levels in symptomatic xylem

Gene ID	Description	LogFC	LogCPM
MD14G1073400	ARM repeat superfamily protein	6.47	5.62
MD07G1102500	–	6.02	5.32
MD15G1425900	ATPase E1-E2 type family protein/haloacid dehalogenase-like hydrolase family protein	5.96	5.28
MD11G1214800	GDSL-like lipase/acylhydrolase superfamily protein	5.78	5.20
MD10G1106500	glycosyl hydrolase family 10 protein	5.72	5.15
MD07G1172700	PAL1B*	5.38	14.69
MD08G1186400	exordium like-2	5.35	5.67
MD00G1144300	–	5.31	4.92
MD01G1106900	PAL1A*	5.28	13.51
MD16G1201800	ATPase E1-E2 type family protein/haloacid dehalogenase-like hydrolase family protein	5.23	4.90
MD02G1005300	FAD/NAD(P)-binding oxidoreductase family protein	5.09	4.82
MD06G1047600	expansin-like B1	5.08	4.84
MD11G1218000	non-coding RNA	4.97	4.79
MD03G1010800	–	4.72	4.69
MD15G1345400	eukaryotic aspartyl protease family protein	4.39	5.12
MD01G1196100	C-repeat/DRE binding factor 2	4.35	5.74
MD12G1016200	nucleotide-diphospho-sugar transferases superfamily protein	4.34	5.40
MD15G1372400	phosphate-responsive 1 family protein	4.15	6.03
MD05G1143500	hydroxy methylglutaryl CoA reductase 1	4.13	5.54
MD09G1039300	zinc finger (AN1-like) family protein	4.07	5.58
MD17G1234700	pentatricopeptide repeat (PPR) superfamily protein	4.05	5.98
MD04G1210300	phosphoglycerate mutase family protein	4.04	5.22
MD07G1126500	BON association protein 2	3.97	5.68
MD12G1038600	NDR1/HIN1-like 3	3.78	6.63

The top 24 genes that had the largest fold change in abundance. Both log fold change (FC) and log counts per million (CPM) are expressed in terms of symptomatic xylem relative to uninfected xylem. PAL1A and PAL1B are denoted by *. See also Tables S4 and S5.

more suitable candidate for the site of catechin biosynthesis because they are known to have a more complex metabolism, and while they form lignified secondary cell walls, this process appears unaffected by ARWV infection (Figure 1).

Symptomatic xylem exhibited a 17-fold reduction in sinapaldehyde together with a significant reduction in syringic acid 4-O hexoside (Figure S3). Reductions in sinapaldehyde and related derivatives are consistent with the lower abundance of COMT1 proteins in symptomatic xylem (Table 1) and the reduction of β-O-4 linkages within syringyl subunits in symptomatic xylem (Figure S1F). Although alterations in COMT1 may alter the lignin subunit composition, it is unlikely to substantially reduce total lignin accumulation.

Downregulation of PAL is a result of virus-induced RNA interference

ARWV possesses only three open reading frames (ORFs) encoding well-characterized viral proteins unlikely to cause specific reductions in PAL.[5] In several cases, it has been demonstrated that viral symptoms are a result of small interfering RNAs (siRNAs) generated from the virus being homologous to a gene from the host plant that results in the targeting and downregulation of the gene as part of the plant's defense against the virus.[30,31] To explore this possibility, we sequenced sRNAs from ARWV-infected and -uninfected grafts, mapped all the sRNA sequences against the ARWV genome, and then remapped them against the apple transcriptome. We identified 54 sRNA sequences that were found in all 4 viral samples with more than 10 reads per sample (Table S4). The psRNATarget identified 6,888 potential target sites in the apple transcriptome; however, only one sRNA sequence identified 2 *PAL* genes as potential target sites, albeit with the lowest confidence score and involved only in the inhibition of translation. Consequently, there is no evidence to suggest that siRNAs generated from ARWV are responsible for the decrease in *PAL* expression.

Virus-activated small interfering RNAs (vasiRNAs) are part of the plant's responses to viral infection.[32] These vasiRNAs are generated from several plant genes. vasiRNAs cause widespread silencing of host genes[32] and are considered to form part of the host's broad-based antiviral defense. To investigate

vasiRNAs, we mapped the sRNAs directly to the apple genome and identified siRNAs mapping to 125 plant genes that were significantly upregulated in infected tissue (Tables 2 and S5). siRNAs homologous to the 3 *PAL* genes, which we had previously identified as being downregulated during infection (Table 1), exhibited some of the biggest changes in siRNA abundance and were 220 times more abundant in RNA from ARWV-infected xylem (Tables 2 and S5). These vasiRNAs are the likely cause of PAL suppression. Why PAL would be targeted in this way is unclear. It is possible that some of the novel phenylpropanoid compounds generated may have antiviral properties.

Aside from *PAL*, no other lignin biosynthesis genes or transcripts are downregulated (Table 1), and only *PAL* transcripts are associated with an increased mapping of vasiRNAs genes (Table 2). Consequently, the alteration in lignin appears to be initiated by vasiRNAs that decrease levels of PAL mRNA and protein. Changes in the expression and abundance of other mRNAs and proteins associated with lignin and phenylpropanoid biosynthesis are likely to be the result of a feedback mechanism resulting from the decrease in lignin biosynthesis and/or the accumulation of soluble phenolics.

A biomimetic approach to biomass improvement

We are not aware of any other virus that can cause symptoms primarily by altering a major metabolic pathway, such as lignin biosynthesis. Although there has been considerable success in engineering lignin to enhance sugar release,[8,11,12,33,34] mutants with severe lignin alterations can lead to growth abnormalities,[6] and this is the case when the PAL gene function is knocked out.[35] The normal growth of ARWV-infected apple trees is likely a result of the lignin alterations being largely confined to the xylem fibers, as xylem vessels retain a distinct lumen suitable for water transport and do not exhibit the severely collapsed vessels exhibited in some plants with altered lignification.[16] Similarly, the normal plant yield can be maintained when lignin biosynthesis is reduced in fibers only as opposed to all cell types.[36]

The variable nature of ARW disease means that infected trees are of little value in generating improved wood. However, revealing the molecular mechanism underlying ARWV symptoms offers an opportunity to improve the woody properties of commercially important trees by effectively copying the mechanism by which the virus induces symptoms. Using RNAi or CRISPR to downregulate *PAL* should reproduce the ARWV symptoms in woody trees. Further support for the feasibility of this approach comes from a study in poplar that revealed that *PAL* downregulation using RNAi gave the largest reductions in lignin with only limited impact on growth.[7]

Finally, widespread genetic engineering of many plants is limited by regulatory hurdles and public opposition, and this appears particularly true for trees.[13,14] It is apparent, however, that technologies considered as new and under regulatory oversight exhibit similarities to events considered to occur naturally. For example, the genome sequence of sweet potato revealed a T-DNA insertion resulting from a historic Agrobacterium infection. This insertion was limited to domesticated varieties and presumably confers some trait that was beneficial for domestication.[37] Here, we show that the mechanism of siRNAs interference used by the virus shares many similarities to mechanisms of RNAi and antisense that have been used more recently to genetically engineer lignin.[7]

A long history of safe use is an important criterion used to assess the risk associated with any novel plant variety. ARWV is now largely, if not totally, eliminated from commercial apple trees; however, an extensive survey in the UK during the 1950s when ARWV was widespread revealed that in some cases, over 50% of apple trees sampled were infected with ARWV.[38] Since the disease was present across the globe for several decades,[39] even conservative estimates would suggest that many thousands of infected apple trees were propagated, and millions of apples from ARWV-infected trees were eaten with no known adverse health or environmental consequences despite the siRNA-induced alterations in lignin caused by the plant's response to the virus.

STAR★METHODS

Detailed methods are provided in the online version of this paper and include the following:

- KEY RESOURCES TABLE
- RESOURCE AVAILABILITY
  - Lead contact
  - Materials availability
  - Data and code availability
- EXPERIMENTAL MODEL AND SUBJECT DETAILS
  - Plant material and growth conditions
- METHOD DETAILS
  - Bright field microscopy
  - Isolating symptomatic xylem
  - Electron microscopy
  - Cell wall sugar analysis
  - Digestibility assay
  - Raman imaging
  - Lignin content
  - Solid-state nuclear magnetic resonance
  - Metabolomic analysis
  - RNA-Sequencing
  - Proteomic analysis
- QUANTIFICATION AND STATISTICAL ANALYSIS

SUPPLEMENTAL INFORMATION

Supplemental information can be found online at https://doi.org/10.1016/j.cub.2022.06.005.

A video abstract is available at https://doi.org/10.1016/j.cub.2022.06.005#mmc6.

ACKNOWLEDGMENTS

The authors would like to acknowledge the support of the Leverhulme Trust (grant RPG-2014-300) for support of H.A. and the BBSRC (grant BB/P01013X/1) for the support of M.K. They also acknowledge the local Galaxy service provided by Peter Briggs at the Bioinformatics Core Facility and IT services, proteomic analysis provided by staff at the Biological Mass Spectrometry Facility (RRID: SCR_020987), RNA-seq analysis provided by the Genomic Technologies and Bioinformatics facilities and the Bioinformatics core facility, and electron microscopy provided by the Electron Microscopy Facility in the Faculty of Biology, Medicine and Health at the University of Manchester.

AUTHOR CONTRIBUTIONS

Conceptualization, S.R.T. and H.A.; methodology, H.A., G.G., L.D.G., and A.P.D.; investigation, H.A., G.G., K.M., L.D.G., A.P.D., M.K., L.Z., and A.E.;

writing – original draft, H.A. and S.R.T.; writing – review & editing, H.A., S.R.T., and W.B.; funding acquisition, S.R.T.; supervision, S.R.T., W.B., S.J.M.-M., and C.C.

DECLARATION OF INTERESTS

The authors declare no competing interests.

Received: February 7, 2022
Revised: March 29, 2022
Accepted: June 1, 2022
Published: June 21, 2022

REFERENCES

1. Somerville, C., Youngs, H., Taylor, C., Davis, S.C., and Long, S.P. (2010). Feedstocks for lignocellulosic biofuels. Science *329*, 790–792.
2. Zhang, J., Cai, D., Qin, Y., Liu, D., and Zhao, X. (2020). High value-added monomer chemicals and functional bio-ased materials derived from polymeric components of lignocellulose by organosolv fractionation. Biofuels Bioprod. Biorefining *14*, 371–401.
3. Xiao, S., Chen, C., Xia, Q., Liu, Y., Yao, Y., Chen, Q., Hartsfield, M., Brozena, A., Tu, K., Eichhorn, S.J., et al. (2021). Lightweight, strong, moldable wood via cell wall engineering as a sustainable structural material. Science *374*, 465–471.
4. Beakbane, A.B., and Thompson, E.C. (1945). Abnormal lignification in the wood of some apple trees. Nature *156*, 145–146.
5. Rott, M.E., Kesanakurti, P., Berwarth, C., Rast, H., Boyes, I., Phelan, J., and Jelkmann, W. (2018). Discovery of negative-sense RNA viruses in trees infected with apple rubbery wood disease by next-generation sequencing. Plant Dis. *102*, 1254–1263.
6. Chen, F., and Dixon, R.A. (2007). Lignin modification improves fermentable sugar yields for biofuel production. Nat. Biotechnol. *25*, 759–761.
7. Wang, J.P., Matthews, M.L., Williams, C.M., Shi, R., Yang, C., Tunlaya-Anukit, S., Chen, H.-C., Li, Q., Liu, J., Lin, C.-Y., et al. (2018). Improving wood properties for wood utilization through multi-omics integration in lignin biosynthesis. Nat. Commun. *9*, 1579.
8. Wilkerson, C.G., Mansfield, S.D., Lu, F., Withers, S., Park, J.-Y., Karlen, S.D., Gonzales-Vigil, E., Padmakshan, D., Unda, F., Rencoret, J., and Ralph, J. (2014). Monolignol ferulate transferase introduces chemically labile linkages into the lignin backbone. Science *344*, 90–93.
9. Menon, V., and Rao, M. (2012). Trends in bioconversion of lignocellulose: biofuels, platform chemicals & biorefinery concept. Prog. Energy Combust. Sci. *38*, 522–550.
10. Marriott, P.E., Gómez, L.D., and McQueen-Mason, S.J. (2016). Unlocking the potential of lignocellulosic biomass through plant science. New Phytol. *209*, 1366–1381.
11. de Meester, B., Madariaga Calderón, B., de Vries, L., Pollier, J., Goeminne, G., van Doorsselaere, J., Chen, M., Ralph, J., Vanholme, R., and Boerjan, W. (2020). Tailoring poplar lignin without yield penalty by combining a null and haploinsufficient CINNAMOYL-CoA REDUCTASE2 allele. Nat. Commun. *11*, 5020.
12. Chanoca, A., de Vries, L., and Boerjan, W. (2019). Lignin engineering in forest trees. Front. Plant Sci. *10*, 912.
13. Strauss, S.H., Boerjan, W., Chiang, V., Costanza, A., Coleman, H., Davis, J.M., Lu, M.-Z., Mansfield, S.D., Merkle, S., Myburg, A., et al. (2019). Certification for gene-edited forests. Science *365*, 767–768.
14. Strauss, S.H., Costanza, A., and Séguin, A. (2015). BIOTECHNOLOGY. Genetically engineered trees: paralysis from good intentions. Science *349*, 794–795.
15. Patten, A.M., Cardenas, C.L., Cochrane, F.C., Laskar, D.D., Bedgar, D.L., Davin, L.B., and Lewis, N.G. (2005). Reassessment of effects on lignification and vascular development in the irx4 Arabidopsis mutant. Phytochemistry *66*, 2092–2107.
16. Jones, L., Ennos, A.R., and Turner, S.R. (2001). Cloning and characterization of irregular xylem4 (irx4): a severely lignin-deficient mutant of Arabidopsis. Plant J. *26*, 205–216.
17. Wierzbicki, M.P., Maloney, V., Mizrachi, E., and Myburg, A.A. (2019). Xylan in the middle: understanding xylan biosynthesis and its metabolic dependencies toward improving wood fiber for industrial processing. Front. Plant Sci. *10*, 176.
18. Sondheimer, E., and Simpson, W.G. (1962). Lignin abnormalities of rubbery apple wood. Can. J. Biochem. Physiol. *40*, 841–846.
19. Djajadi, D.T., Jensen, M.M., Oliveira, M., Jensen, A., Thygesen, L.G., Pinelo, M., Glasius, M., Jørgensen, H., and Meyer, A.S. (2018). Lignin from hydrothermally pretreated grass biomass retards enzymatic cellulose degradation by acting as a physical barrier rather than by inducing nonproductive adsorption of enzymes. Biotechnol. Biofuels *11*, 85.
20. Liang, M., Davis, E., Gardner, D., Cai, X., and Wu, Y. (2006). Involvement of AtLAC15 in lignin synthesis in seeds and in root elongation of *Arabidopsis*. Planta *224*, 1185–1196.
21. Shigeto, J., Itoh, Y., Hirao, S., Ohira, K., Fujita, K., and Tsutsumi, Y. (2015). Simultaneously disrupting *AtPrx2*, *AtPrx25* and *AtPrx71* alters lignin content and structure in *Arabidopsis* stem. J. Integr. Plant Biol. *57*, 349–356.
22. Fernández-Pérez, F., Pomar, F., Pedreño, M.A., and Novo-Uzal, E. (2015). The suppression of *AtPrx52* affects fibers but not xylem lignification in *Arabidopsis* by altering the proportion of syringyl units. Physiol. Plant. *154*, 395–406.
23. Leplé, J.C., Dauwe, R., Morreel, K., Storme, V., Lapierre, C., Pollet, B., Naumann, A., Kang, K.Y., Kim, H., Ruel, K., et al. (2007). Downregulation of cinnamoyl-coenzyme A reductase in poplar: multiple-level phenotyping reveals effects on cell wall polymer metabolism and structure. Plant Cell *19*, 3669–3691.
24. Vanholme, R., Storme, V., Vanholme, B., Sundin, L., Christensen, J.H., Goeminne, G., Halpin, C., Rohde, A., Morreel, K., and Boerjan, W. (2012). A systems biology view of responses to lignin biosynthesis perturbations in *Arabidopsis*. Plant Cell *24*, 3506–3529.
25. Vanholme, R., Demedts, B., Morreel, K., Ralph, J., and Boerjan, W. (2010). Lignin biosynthesis and structure. Plant Physiol. *153*, 895–905.
26. Rohde, A., Morreel, K., Ralph, J., Goeminne, G., Hostyn, V., de Rycke, R., Kushnir, S., van Doorsselaere, J., Joseleau, J.P., Vuylsteke, M., et al. (2004). Molecular phenotyping of the *pal1* and *pal2* mutants of *Arabidopsis thaliana* reveals far-reaching consequences on phenylpropanoid, amino acid, and carbohydrate metabolism. Plant Cell *16*, 2749–2771.
27. Lynch, J.H., Orlova, I., Zhao, C., Guo, L., Jaini, R., Maeda, H., Akhtar, T., Cruz-Lebron, J., Rhodes, D., Morgan, J., et al. (2017). Multifaceted plant responses to circumvent Phe hyperaccumulation by downregulation of flux through the shikimate pathway and by vacuolar Phe sequestration. Plant J. *92*, 939–950.
28. Maeda, H., Shasany, A.K., Schnepp, J., Orlova, I., Taguchi, G., Cooper, B.R., Rhodes, D., Pichersky, E., and Dudareva, N. (2010). RNAi suppression of *Arogenate Dehydratase1* reveals that phenylalanine is synthesized predominantly via the arogenate pathway in petunia petals. Plant Cell *22*, 832–849.
29. Yoo, H., Widhalm, J.R., Qian, Y., Maeda, H., Cooper, B.R., Jannasch, A.S., Gonda, I., Lewinsohn, E., Rhodes, D., and Dudareva, N. (2013). An alternative pathway contributes to phenylalanine biosynthesis in plants via a cytosolic tyrosine:phenylpyruvate aminotransferase. Nat. Commun. *4*, 2833.
30. Smith, N.A., Eamens, A.L., and Wang, M.-B. (2011). Viral small interfering RNAs target host genes to mediate disease symptoms in plants. PLoS Pathog. *7*, e1002022.
31. Shimura, H., Pantaleo, V., Ishihara, T., Myojo, N., Inaba, J., Sueda, K., Burgyán, J., and Masuta, C. (2011). A viral satellite RNA induces yellow symptoms on tobacco by targeting a gene involved in chlorophyll biosynthesis using the RNA silencing machinery. PLoS Pathog. *7*, e1002021.
32. Cao, M., Du, P., Wang, X., Yu, Y.-Q., Qiu, Y.-H., Li, W., Gal-On, A., Zhou, C., Li, Y., and Ding, S.-W. (2014). Virus infection triggers widespread

silencing of host genes by a distinct class of endogenous siRNAs in *Arabidopsis*. Proc. Natl. Acad. Sci. USA *111*, 14613–14618.

33. van Acker, R., Vanholme, R., Storme, V., Mortimer, J.C., Dupree, P., and Boerjan, W. (2013). Lignin biosynthesis perturbations affect secondary cell wall composition and saccharification yield in *Arabidopsis thaliana*. Biotechnol. Biofuels *6*, 46.
34. de Meester, B., de Vries, L., Özparpucu, M., Gierlinger, N., Corneillie, S., Pallidis, A., Goeminne, G., Morreel, K., de Bruyne, M., de Rycke, R., et al. (2018). Vessel-specific reintroduction of cinnamoyl-CoA REDUCTASE1 (CCR1) in dwarfed *ccr1* mutants restores vessel and xylary fiber integrity and increases biomass. Plant Physiol. *176*, 611–633.
35. Huang, J., Gu, M., Lai, Z., Fan, B., Shi, K., Zhou, Y.-H., Yu, J.-Q., and Chen, Z. (2010). Functional analysis of the Arabidopsis *PAL* gene family in plant growth, development, and response to environmental stress. Plant Physiol. *153*, 1526–1538.
36. Gui, J., Lam, P.Y., Tobimatsu, Y., Sun, J., Huang, C., Cao, S., Zhong, Y., Umezawa, T., and Li, L. (2020). Fibre-specific regulation of lignin biosynthesis improves biomass quality in Populus. New Phytol. 226, 1074–1087.
37. Kyndt, T., Quispe, D., Zhai, H., Jarret, R., Ghislain, M., Liu, Q., Gheysen, G., and Kreuze, J.F. (2015). The genome of cultivated sweet potato contains *Agrobacterium* T-DNAs with expressed genes: an example of a naturally transgenic food crop. Proc. Natl. Acad. Sci. USA *112*, 5844–5849.
38. Posnette, A., and Cropley, R. (1954). Distribution of rubbery wood virus in apple varieties and rootstocks (Report of East Malling Research Station), pp. 150–153.
39. Chamberlain, E.E., Atkinson, J.D., Wood, G.A., and Hunter, J.A. (1971). Apple rubbery wood virus. N. Z. J. Agric. Res. *14*, 707–719.
40. Dai, X., Zhuang, Z., and Zhao, P.X. (2018). psRNATarget: a plant small RNA target analysis server (2017 release). Nucleic Acids Res. *46*, W49–W54.
41. Love, M.I., Huber, W., and Anders, S. (2014). Moderated estimation of fold change and dispersion for RNA-seq data with DESeq2. Genome Biol. *15*, 550.
42. Dobin, A., Davis, C.A., Schlesinger, F., Drenkow, J., Zaleski, C., Jha, S., Batut, P., Chaisson, M., and Gingeras, T.R. (2013). STAR: ultrafast universal RNA-seq aligner. Bioinformatics *29*, 15–21.
43. Afgan, E., Baker, D., Batut, B., van den Beek, M., Bouvier, D., Čech, M., Chilton, J., Clements, D., Coraor, N., Grüning, B.A., et al. (2018). The Galaxy platform for accessible, reproducible and collaborative biomedical analyses: 2018 update. Nucleic Acids Res. *46*, W537–W544.
44. Robinson, M.D., McCarthy, D.J., and Smyth, G.K. (2010). edgeR: a Bioconductor package for differential expression analysis of digital gene expression data. Bioinformatics *26*, 139–140.
45. Langmead, B., Trapnell, C., Pop, M., and Salzberg, S.L. (2009). Ultrafast and memory-efficient alignment of short DNA sequences to the human genome. Genome Biol. *10*, R25.
46. Kim, D., Paggi, J.M., Park, C., Bennett, C., and Salzberg, S.L. (2019). Graph-based genome alignment and genotyping with HISAT2 and HISAT-genotype. Nat. Biotechnol. *37*, 907–915.
47. Anders, S., Pyl, P.T., and Huber, W. (2015). HTSeq—a Python framework to work with high-throughput sequencing data. Bioinformatics *31*, 166–169.
48. Bradley, S., and Garner, R. (2013). The Grafter's Handbook (Revised & Updated Edition (Mitchell Beazley)).
49. Grzeskowiak, V., Sassus, F., and Fournier, M. (1996). Coloration macroscopique, retraits longitudinaux de maturation et de séchage Du Bois de tension du peuplier (Populus × euramericana cv I.214). Ann. For. Sci. *53*, 1083–1097.
50. Zhang, B., and Zhou, Y. (2017). In Carbohydrate composition analysis in xylem, Xylem., ed. (Humana Press), pp. 213–222.
51. Kumar, M., and Turner, S. (2015). Protocol: a medium-throughput method for determination of cellulose content from single stem pieces of *Arabidopsis thaliana*. Plant Methods *11*, 46.
52. Brown, D.M., Zeef, L.A.H., Ellis, J., Goodacre, R., and Turner, S.R. (2005). Identification of novel genes in Arabidopsis involved in secondary cell wall formation using expression profiling and reverse genetics. Plant Cell *17*, 2281–2295.
53. Gomez, L.D., Whitehead, C., Barakate, A., Halpin, C., and McQueen-Mason, S.J. (2010). Automated saccharification assay for determination of digestibility in plant materials. Biotechnol. Biofuels *3*, 23.
54. Gierlinger, N., and Schwanninger, M. (2007). The potential of Raman microscopy and Raman imaging in plant research. Spectroscopy *21*, 69–89.
55. Barnes, W.J., and Anderson, C.T. (2017). Acetyl bromide soluble lignin (ABSL) assay for total lignin quantification from plant biomass. Bio Protoc. *7*, e2149.
56. Foster, C.E., Martin, T.M., and Pauly, M. (2010). Comprehensive compositional analysis of plant cell walls (Lignocellulosic biomass) part I: lignin. J. Vis. Exp. *1745*.
57. Kumar, M., Mishra, L., Carr, P., Pilling, M., Gardner, P., Mansfield, S.D., and Turner, S. (2018). Exploiting cellulose synthase (CESA) class specificity to probe cellulose microfibril biosynthesis. Plant Physiol. *177*, 151–167.
58. Dupree, R., Simmons, T.J., Mortimer, J.C., Patel, D., Iuga, D., Brown, S.P., and Dupree, P. (2015). Probing the molecular architecture of *Arabidopsis thaliana* secondary cell walls using two- and three-dimensional (13)C solid state nuclear magnetic resonance spectroscopy. Biochemistry *54*, 2335–2345.
59. Bardet, M., Foray, M.F., and Trân, Q.-K. (2002). High-resolution solid-state CPMAS NMR study of archaeological woods. Anal. Chem. *74*, 4386–4390.
60. Santoni, I., Callone, E., Sandak, A., Sandak, J., and Dirè, S. (2015). Solid state NMR and IR characterization of wood polymer structure in relation to tree provenance. Carbohydr. Polym. *117*, 710–721.
61. Edmunds, C.W., Peralta, P., Kelley, S.S., Chiang, V.L., Sharma-Shivappa, R.R., Davis, M.F., Harman-Ware, A.E., Sykes, R.W., Gjersing, E., Cunningham, M.W., et al. (2017). Characterization and enzymatic hydrolysis of wood from transgenic *Pinus taeda* engineered with syringyl lignin or reduced lignin content. Cellulose *24*, 1901–1914.
62. Schenk, S., and Schikora, A. (2015). Lignin extraction and quantification, a tool to monitor defense reaction at the plant cell wall level. Bio Protoc. *5*, e1430.
63. Ainsworth, E.A., and Gillespie, K.M. (2007). Estimation of total phenolic content and other oxidation substrates in plant tissues using Folin–Ciocalteu reagent. Nat. Protoc. *2*, 875–877.
64. Desmet, S., Saeys, Y., Verstaen, K., Dauwe, R., Kim, H., Niculaes, C., Fukushima, A., Goeminne, G., Vanholme, R., Ralph, J., et al. (2021). Maize specialized metabolome networks reveal organ-preferential mixed glycosides. Comput. Struct. Biotechnol. J. *19*, 1127–1144.
65. Morreel, K., Saeys, Y., Dima, O., Lu, F., van de Peer, Y., Vanholme, R., Ralph, J., Vanholme, B., and Boerjan, W. (2014). Systematic structural characterization of metabolites in *Arabidopsis* via candidate substrate-product pair networks. Plant Cell *26*, 929–945.
66. Dührkop, K., Shen, H., Meusel, M., Rousu, J., and Böcker, S. (2015). Searching molecular structure databases with tandem mass spectra using CSI:FingerID. Proc. Natl. Acad. Sci. USA *112*, 12580–12585.
67. Dührkop, K., Fleischauer, M., Ludwig, M., Aksenov, A.A., Melnik, A.V., Meusel, M., Dorrestein, P.C., Rousu, J., and Böcker, S. (2019). SIRIUS 4: a rapid tool for turning tandem mass spectra into metabolite structure information. Nat. Methods *16*, 299–302.
68. Daccord, N., Celton, J.-M., Linsmith, G., Becker, C., Choisne, N., Schijlen, E., van de Geest, H., Bianco, L., Micheletti, D., Velasco, R., et al. (2017). High-quality *de novo* assembly of the apple genome and methylome dynamics of early fruit development. Nat. Genet. *49*, 1099–1106.

STAR★METHODS

KEY RESOURCES TABLE

REAGENT or RESOURCE	SOURCE	IDENTIFIER
Chemicals, peptides, and recombinant proteins		
Iodine	Millipore Sigma	Cat#207772
Potassium-iodide	Millipore Sigma	Cat#221945
Sodium hydroxide	Fisher	Cat#AAA1603736
Zinc Chloride	Fisher	Cat#AAA1628136
Toluidine Blue	Millipore Sigma	Cat#89640
Safranin-O	Millipore Sigma	Cat#S2255
Astra Blue	Solmedia Ltd	Cat#DYE001-C
HEPES	Millipore Sigma	Cat#H3375
Anthrone	Millipore Sigma	Cat#319899
Glutaraldehyde	Agar Scientific	R1012 100ml
Paraformaldehyde	Agar Scientific	Cat#R1102 500g
Potassium Hexacyanoferrate (II) trihydrate	Merck	Cat#60279-250G
Osmium Tetroxide 5x5ml ampules at 4%	Agar Scientific	Cat#R1024
Sodium cacodylate	Agar Scientific	R1018 500g
Uranyl acetate	Taab	U001 50g
Medium TAAB LV Resin premix kits medium	Taab	T262
Sodium borohydride	Sigma	213462-25g
Acetyl bromide	Millipore Sigma	Cat#135968
Hydroxylamine Hydrochloride	Millipore Sigma	Cat#159417
Myo-inositol	Millipore Sigma	Cat#I5125
Chloroform	Fisher	Cat#15498679
Formic acid	Millipore Sigma	Cat#F0507
TEAB	Millipore Sigma	Cat#140023
Acetonitrile	Pierce	51101
Methanol	Fisher	M/4000/17
Acetone	Fisher	A/0600/PC17
Ethanol	Fisher	E/0650DF/P17
Ammonia	Fisher	A/3295/PB05
Dichloromethane	Sigma	270997-1L
Cyclohexane	Sigma	34859L
1-methylimidazole	Sigma	336092-100ML
Acetic anhydride	Millipore Sigma	Cat#AX0080
Glacial acetic acid	Fisher	A/0400/PB1
Nitric acid	Fisher	N/2300/PB15
Sulphuric acid	Fisher	S/9160/PB15
Sodium dodecyl sulphate	Sigma	75746-1KG
Glycerol	Fisher	G/0650/08
Dithiothreitol	Thermo	R0862
Arabinose	Sigma	14/112-17
Glucose	Sigma	G/0500/61
Galactose	Sigma	G-0750
Xylose	BD	218110
Mannose	Sigma	11,258-5
Rhamnose	Sigma	17,198-0

(*Continued on next page*)

REAGENT or RESOURCE	SOURCE	IDENTIFIER
Continued		
Gallic acid	Sigma	G-0750
Hydrochloric acid	Fisher	H/1200/PB15 1l
Methanol	Fisher	M/4000/17
Ultra-pure methanol	VWR	85800.290
BCA protein assay reagent	Merck Millipore	71285-3
Amylase	Millipore Sigma	Cat#A3306
Pullulanase	Millipore Sigma	Cat#E2412
Cellulclast/Novozyme 188 enzyme cocktail	University of York	N/A
Iodoacetamide	Sigma	Cat#I1149
Dithiothreitol for proteomics	Fisher	Cat#BP172-5
Triethylammonium bicarbonate	Sigma	Cat#T7408-100mL
0.1% aqueous Formic acid	Merck	Cat#1.59013.2500
Acetonitrile	Fisher	Cat#A955-1
S-TRAP columns	Protifi	Cat#CO2-micro-80
TPCK treated trypsin,	Worthington	Cat#LS003740 100mg
**Deposited data**		
RNA dataset	This study	Array express E-MTAB-11410
sRNA dataset	This study	Array express E-MTAB-11409
Proteomics dataset	This study	ProteomeXchange PXD03134
Metabolomics dataset Raw data	Metabolights	Metabolights project MTBLS4632
**Biological samples**		
Lord Lambourne budwood	East Malling Research Station, Kent, UK	N/A
Lord Lambourne budwood infected with ARWV	East Malling Research Station, Kent, UK	N/A
MM106 2 year 5-7mm rootstock	Frank P Matthews Tree Shop	Cat#106634
**Software and algorithms**		
WITec Project software v5.3	WITec Project software	https://raman.oxinst.com
Progenesis QI software v2.1	Progenesis QI software	https://waters.com
Inkscape 1.1	Inkscape Software	https://inkscape.org/
ChemSketch	ChemSketch	https://www.acdlabs.com/
Progenesis QI for proteomics	Progenesis QI software	https://waters.com
psRNAtarget 2017 release	Dai et al.[40]	https://www.zhaolab.org/psRNATarget/home
DESeq2 v1.18.1	Love et al.[41]	https://bioconductor.org/packages/release/bioc/html/DESeq2.html
STAR v2.4.2	Dobin et al.[42]	https://github.com/alexdobin/STAR/tree/STAR_2.4.2a
Galaxy 20.5 maintained locally.	Afgan et al.[43]	https://usegalaxy.org
EdgeR using the Galaxy wrapper	Robinson et al.[44]	toolshed.g2.bx.psu.edu/repos/iuc/edger/edger/3.24.1+galaxy1
Bowtie1 using the Galaxy wrapper	Langmead et al.[45]	toolshed.g2.bx.psu.edu/repos/devteam/bowtie_wrappers/bowtie_wrapper/1.2.0
HiSat2 using the Galaxy wrapper	Kim et al.[46]	toolshed.g2.bx.psu.edu/repos/iuc/hisat2/hisat2/2.1.0+galaxy5
Htseq-count using the Galaxy wrapper	Anders et al.[47]	toolshed.g2.bx.psu.edu/repos/lparsons/htseq_count/htseq_count/0.9.1
GraphPad Prism v9.0.0	GraphPad Software	https://graphpad.com

RESOURCE AVAILABILITY

Lead contact

Further information and requests for resources, reagents and datasets should be directed to and will be fulfilled by the lead contact, Professor Simon Turner (simon.turner@manchester.ac.uk)

Materials availability

Apple tree material used in this study will be made available upon request without any restriction.

Data and code availability

- RNAseq and sRNAseq datasets have been deposited at ArrayExpress, the proteomic dataset has been deposited at ProteomeXchange, and the metabolomic dataset has been deposited at Metabolights, and are all publicly available as of the date of publication. Accession numbers are listed in the key resources table. All other datatypes in this paper will be shared by the lead contact upon request.
- No code was generated in this study.
- Any additional information required to reanalyze the data reported in this paper is available from the lead contact upon request.

EXPERIMENTAL MODEL AND SUBJECT DETAILS

Plant material and growth conditions

Apple stem material was propagated from 2 uninfected and 2 ARWV-infected Lord Lambourne apple trees, which were provided by the East Malling Research Station, Kent, UK in 1996. Budwood was grafted onto M27 rootstocks using the 'whip and tongue' grafting method[48] in April 2017. Grafts were kept in a glass house at 22°C and one-year old stem material was harvested the following year for analysis.

METHOD DETAILS

Bright field microscopy

Fresh apple stem material was cut between nodes into approximately 1.5-inch segments and then divided longitudinally into smaller, more manageable segments using a razor blade. 10 μm transverse segments were cut using a sliding RM2155 microtome. Cross sections were briefly stained with the Herzberg stain [6% iodine, 14% potassium-iodide dissolved in a saturated $ZnCL_2$ solution (w/v)] directly on the slide[49] or 0.05% Toluidine blue (w/v). For basic Fuchsin/Astra-blue staining, sections were sequentially stained with 1% basic Fuchsin dissolved in 50% ethanol, followed by 10% Astra Blue pH 2.5 dissolved in 100% ethanol. Stained sections were imaged immediately using a Leica 5500 microscope with a Spot RT3 camera. Sections shown in Figure 1 are representative of more than 400 toluidine blue stained sections from uninfected and ARWV-infected trees taken over 4 successive growing seasons from more than 100 different grafts. For Herzberg staining, more than 100 ARWV-infected and 50 uninfected samples were sectioned and for Astra blue staining, 4 symptomatic and 4 uninfected sections were examined. Electron micrographs are representatives of sections from blocks from more than 10 uninfected and 10 symptomatic samples.

Isolating symptomatic xylem

To dissect symptomatic xylem from asymptomatic xylem, branches were firstly cut between nodes into smaller segments. Each cut branch segment was then sectioned on either side and stained with 0.05% Toluidine blue to assess the distribution of symptoms along the internode. Using the images as a guide, symptomatic regions were isolated from the branch segment with a razor blade and the ends of the branch segment were then re-sectioned and stained to confirm the removal of all the asymptomatic regions. If both sections showed only symptomatic regions, the whole branch fragment was regarded as entirely symptomatic. For nucleic acid and metabolite extraction, sample were enriched for newly differentiating xylem by scraping from symptomatic regions following the removal of the bark.

Electron microscopy

Segments cut from fresh apple stem material were immediately placed in fixative [2% glutaraldehyde, 2% paraformaldehyde dissolved in 0.1 M HEPES buffer, pH 7.2, (v/v)], vacuum infiltrated and then incubated overnight at 4°C. Fixed stem material was stained for two hours in 2% reduced osmium dissolved in 0.1 M sodium cacodylate, pH 7.2 (v/v), followed by staining with 1% uranyl acetate overnight. Stained segments were gradually dehydrated with increasing concentrations of ethanol (25%, 50%, 70%, 90%, 100%), followed by acetone, for one hour each. Segments were progressively infiltrated with increasing concentrations of Medium TAAB resin, mixed in acetone (25%, 50%, 75%,100%) over the course of three days and polymerised at 60°C for 5 days. Ultra-thin sections

were cut with an ultracut microtome using a DiATOME Ultra 45° knife and mounted on 3.05 nm Athene nickel with a 0.3% formvar coating. Grids were examined on a Tecnai T12 BioTWIN transmission electron microscope and photographed with an Orius CCD SC1000 camera.

Cell wall sugar analysis

Uninfected, asymptomatic and symptomatic xylem segments were sliced thinly with a razor blade, flash frozen in liquid nitrogen and stored at -80°C. Frozen material was freeze dried for 24 hours and then ground into a fine powder using a Tissuelyser II for 10 minutes at 1/24 Hz. Cell walls were isolated and de-starched according to Zhang and Zhou.[50] 100 mg of ground freeze-dried material was mixed with 70% ethanol, 1:1 chloroform/methanol and acetone sequentially and the remaining acetone was left to evaporate overnight in a fume hood. Starch was removed by re-suspending the cell wall material in 5 ml 0.1 M NaOH buffer, pH 5.0, and heating at 80°C for 20 minutes. After cooling on ice, 20 μl amylase solution (250 μg/ml) and 5 μl pullulanase solution was added to re-suspended cell wall material and incubated at 37°C overnight. To stop the reaction, cell walls were heated at 100°C for 10 minutes and centrifuged at 1,300 x g for 10 minutes to pellet the de-starched cell walls. Samples were washed three times with distilled water and once with acetone, leaving the acetone to evaporate.

Crystalline cellulose content was quantified using the Updegraff method using 6 uninfected, 8 asymptomatic and 8 symptomatic samples.[51] Hemicellulose was removed from approximately 10 mg cell wall material by adding Updegraff reagant (glacial acetic acid: nitric acid: water, 8:1:2) and heating samples at 100°C for 30 minutes. Samples were centrifuged for 10 minutes and the supernatant was discarded. Samples were washed with distilled water and acetoen sequentially leaving the remainder of the acetone to evaporate overnight in the fume hood. Crystalline cellulose was solubilised into glucose by incubating cell walls with 67% sulphuric acid, overnight on a shaker. To measure glucose content, 0.3% anthrone dissolved in sulphuric acid (v/v) was added to acidified samples or glucose standards, and samples were heated at 100°C for 5 minutes. Absorbance was measured at 620 nm on a spectrophotometer and cellulose content was calculated from a glucose standard curve.

Non-cellulosic monosaccharides were measured with gas chromatography using 8 uninfected and 7 symptomatic samples.[52] 500 μl 1 M sulphuric acid was added to approximately 10 mg of cell wall material and incubated at 121 °C for 1 hour. Myo-inositol was added as an internal control. Once cooled, samples were centrifuged for 5 minutes and 250 μl of the supernatant was added to 100 μl 9 M NH_3, followed by 1 ml 2% $NaBH_4$ dissolved in DMSO (w/v), mixed thoroughly and heated at 40°C for 90 minutes. 250 μl glacial acetic acid was then added, followed by 250 μl 1-methylimidazole, 4 ml of acetic anhydride, and then briefly mixed. 8 ml of double-distilled water was added, and the samples were mixed until the precipitate dissolved. 1.5 ml dichloromethane was then added and thoroughly mixed, before incubating at 4°C overnight to extract acetylated alditol derivatives. The lower phase was recovered and heated at 55 °C for 45 minutes, or until the filtrate had evaporated. 250 μl dichloromethane and 1 ml double distilled water was added and vortexed vigorously, to extract hydrophilic compounds into the aqueous phase. When cooled, approximately 200 μl of the lower phase were analysed on a Supelco SP-2330 column attached to an Agilent 6850 GC instrument. Peaks were assigned by running 20 mg/ml sugar standards (rhamnose, arabinose, xylose, mannose, glucose, galactose and myo-inositol). Monosaccharide content was calculated by dividing the area under each sugar peak with the correction factor determined by the myo-inositol standards.

Digestibility assay

Stem material from 6 uninfected and 6 infected grafts were partially ground with a mortar and pestle under liquid nitrogen and then freeze dried for 48 hours. For each sample, 100 mg was pre-treated with alkali (0.5 M NaOH for 30 minutes at 90°C), a weak acid (0.5% H_2SO_4 for 30 minutes at 90°C), or it received no pre-treatment. All samples were hydrolysed with a Cellulclast:Novozyme 188 enzyme cocktail over 18 hours using an automated platform according to Gomez et al.[53] Digestibility was calculated as the adjusted nmol reducing sugars / mg material.

Raman imaging

Thin sections of ~20 μm were cut on a microtome, placed on a glass slide with a drop of water and cover slip, and the edges were sealed to prevent evaporation. Raman 2-dimensional spectral maps were acquired with a confocal Witec spectrometer equipped with a 514.5 nm (2.41 eV) laser using a backscattering configuration. A 20μm × 20μm area of cells was selected for Raman scanning. A 100× objective was used, giving a laser spot size of ~200 nm, and the spectral resolution was $\simeq$ 1 cm^{-1}. Spectral images were processed and analysed using WITec Project software v5.3. Raman maps were computed from the spectra by using a sum filter, integrating over the wavenumber range 1540-1780 cm^{-1} for lignin, and 2830-3040 cm^{-1} for cellulose.[54] The images shown are from a region that was deemed to be representative based on screening of toluidine blue stained sections.

Lignin content

Soluble lignin content was quantified from 5 mg de-starched cell wall material using an acetyl-bromide assay, based on the method described by Barnes and Anderson.[55] Approximately 5 mg cell wall material was incubated with 1 ml 25% acetyl bromide dissolved in acetic acid (v/v) and heated for 4 hours at 50°C. After cooling on ice, 5 ml glacial acetic acid was added and vortexed thoroughly. Residual cell wall material was left to settle overnight at room temperature. 300 μl of the acetyl-bromide solution was mixed with 400 μl 1.5 N NaOH and 300 μl freshly made 0.5 M hydroxylamine hydrochloride. Samples were analysed in triplicate using a microplate reader and the absorbance was recorded at 280 nm. The extinction co-efficient of lignin from *Populus*,[56] 18.21 g^{-1} L cm^{-1}, was

used to calculate the lignin content of apple. Analysis was performed on samples from 6 uninfected, 8 asymptomatic and 8 symptomatic grafts,

Solid-state nuclear magnetic resonance

100 mg de-starched cell wall material from an uninfected and symptomatic graft was analysed using CP-MS ^{13}C-NMR spectroscopy, according to Kumar et al.[57] Briefly, spectra were measured at 100.56 MHz using a Varian VNMRS spectrometer and 4-mm magic angle spinning probe. Cross-polarisation was used to resolve spectra under the following conditions: a 2 second recycle delay, a 3 ms contact time and a spin rate of 10 kHz. The spectra were baseline corrected and normalised so that the total signal for each sample was 100%. In the normalised spectra, peaks were assigned to secondary cell wall polymers based on chemical shifts reported for secondary cell walls in *Arabidopsis*[58] and various tree species.[59–61] The spectra shown is derived from pools of uninfected and symptomatic xylem obtained from at least 3 different trees.

Metabolomic analysis

Soluble phenolics were extracted from 10 mg of stem material according to Schenk and Schikora[62] and measured using the Folin-Ciocalteu method.[63] Approximately 10 mg stem material was incubated in 1 ml 80% methanol for 1 hour. The methanol was replaced with fresh methanol and then sample was pelleted, retaining the superntatnt as the soluble phenolic fraction. To measure the soluble phenolic fraction, 200 μl of Folin-Ciocalteu reagent was added to 100 μl of the supernatant and gallic acid standards. Samples were analysed in triplicate using a microplate reader and the absorbance was recorded at 765 nm and soluble phenolic content was measured from a gallic acid standard curve. For metabolomics analyses, 7 uninfected and 7 infected graft segments were used. After removing the bark, whole xylem segments were flash frozen in liquid nitrogen. Newly differentiating xylem was scraped from frozen xylem segments and then ground into a fine powder using a mortar and pestle with liquid nitrogen. 50 mg of frozen xylem was incubated with 1 ml methanol for 30 minutes at 128 rpm. Samples were centrifuged for 2 minutes and the methanol fraction was dried for two hours using a speed vacuum. The pellet was re-dissolved in 200 μl water / cyclohexane (v/v) and 10 μl of this aqueous phase was analysed by reverse phase UltraHigh Performance Liquid Chromatography (Waters Corporation) with negative ion ElectroSpray Ionization-Quadrupole-Time-Of-Flight-Mass Spectrometry (ESI-Q-TOF; Waters Corporation). Integration and alignment of the m/z features were performed using the Progenesis QI software v2.1. Instrument settings and data processing parameters were as described in Desmet et al.[64] Structural characterization was performed using in-house developed characterization algorithms and databases[64,65] combined with CSI:FingerID.[66,67] Metabolomics data will be deposited at Metabolights (https://www.ebi.ac.uk/metabolights/).

RNA-Sequencing

Total RNA was extracted from the same material used for the metabolomics analysis, using the RNAeasy PlantPower kit, as per the manufacturer's instructions. DNA was removed using the DNAse Max kit and RNA quality and purity was determined using the Agilent 4200 Tapestation. 500 ng of total RNA from 6 uninfected and 6 infected graft segments were sequenced by Illumina HiSeq4000 using 75 bp paired-end sequencing. Raw sequences were mapped to the *Malus x domestica* GDDH13 v1.1 genome and genes were annotated[68] using the RNA-sequencing aligner, STAR.[42] Differential gene expression was calculated using DESeq2 and the threshold significance was set at an FDR-adjusted p-value $\leq$ 0.01 and a log-fold change ± 2. Gene descriptions were added based on the homology of *M. domestica* transcript sequences with the NCBI non-redundant protein database, which was obtained from the online *M. domestica* genome resource (www.rosaceae.org).

Small-RNA was extracted from 140 ng total RNA and the libraries were assembled using the NEBNext Small RNA Library Prep set for Illumina according to the manufacturer's instructions. 1.9 nM small-RNA libraries were sequenced by Illumina NextSeq using 50 nucleotide single end reads. All raw reads were processed using a maintained version of the Galaxy server.[45] The adaptors were then trimmed using Trim Galore (www.bioinformatics.babraham.ac.uk/projects/trim_galore) and size selected for reads of 19-25 nucleotides. Trimmed sequences were mapped to the ARWV genome using Bowtie1,[45] which allowed only exact matches within the seed. Sequences that mapped at least 10 times in each ARWV-infected sample were then used as input for target site analysis, using the psRNAtarget analysis server.[40] Mapping of sRNAs to the GDDH13 v1.1 genome[68] was performed using HiSat2[46] and Htseq-count.[47] Differential expression was calculated using EdgeR,[44] filtering on low counts to include genes that had a minimum of 10 reads in at least 3 samples.

Proteomic analysis

Proteins were extracted from the same material used for metabolomics, with a 1X SDS-based buffer (1.05% SDS, 32.9 mM Tris-HC pH 8, 13.15% glycerol, 50 mM DTT). Extraction buffer was added to ground stem material in a 5:1 ratio, and then samples were heated for 10 minutes at 65°C. Extracts were centrifuged for 10 minutes at 3,000 x g and the liquid phase was retained. 75 μg from each sample was purified and bound to an S-Trap micro column and digested with 5 μg trypsin, according to the manufacturer's instructions. Peptides were eluted with 40 μl 50mM TEAB, 40 μl 0.2% formic acid and 35 μl 50% acetonitrile containing 0.2% formic acid. Pooled elutions were dried for two hours using a HETO SpeedVac. Peptides were analysed on a Q-extractive HF mass spectrometer using ESI-QUAD-TOF. Peptide libraries were normalised to the total amount of protein and peaks were labelled based on the top 3 MS/MS spectra using Progenesis QI for proteomics. Using the MS/MS ions protein IDs were assigned using the *M. domestica* genome v1.1[68] with MASCOT and differential protein abundance was quantified using Progenesis QI for proteomics. The threshold

for significance was set at a minimum 2-fold change that was determined by a minimum of two unique peptides to the protein. Analysis was performed on samples from 5 uninfected and 5 symptomatic samples.

QUANTIFICATION AND STATISTICAL ANALYSIS

Biochemical data were analysed with One-Way ANOVAs combined with Tukey multiple comparison tests in GraphPad Prism v9.0.0 and significance values are indicated on Figures 1 and S1. RNA-seq and sRNA-seq data was analysed using DESeq2 and EdgeR respectively, in RStudio v1.1453. Proteomic data was analysed using Progenesis QI for proteomics and metabolomics data was analysed using Progenesis QI v.2.1 Further information for RNA-seq, sRNA-seq, proteomics and metabolomics analysis is provided in the results and discussion, and STAR methods sections. Sample sizes and confidence intervals for all experiments are indicated in the relevant figures and STAR methods sections. All graphs were generated in GraphPad Prism v9.0.0, chemical structures were created in ChemSketch and biochemical pathways were constructed using InkScape.

Current Biology, Volume *32*

Supplemental Information

Flexible and digestible wood caused by viral-induced alteration of cell wall composition

Holly Allen, Leo Zeef, Kris Morreel, Geert Goeminne, Manoj Kumar, Leonardo D. Gomez, Andrew P. Dean, Axel Eckmann, Cinzia Casiraghi, Simon J. McQueen-Mason, Wout Boerjan, and Simon R. Turner

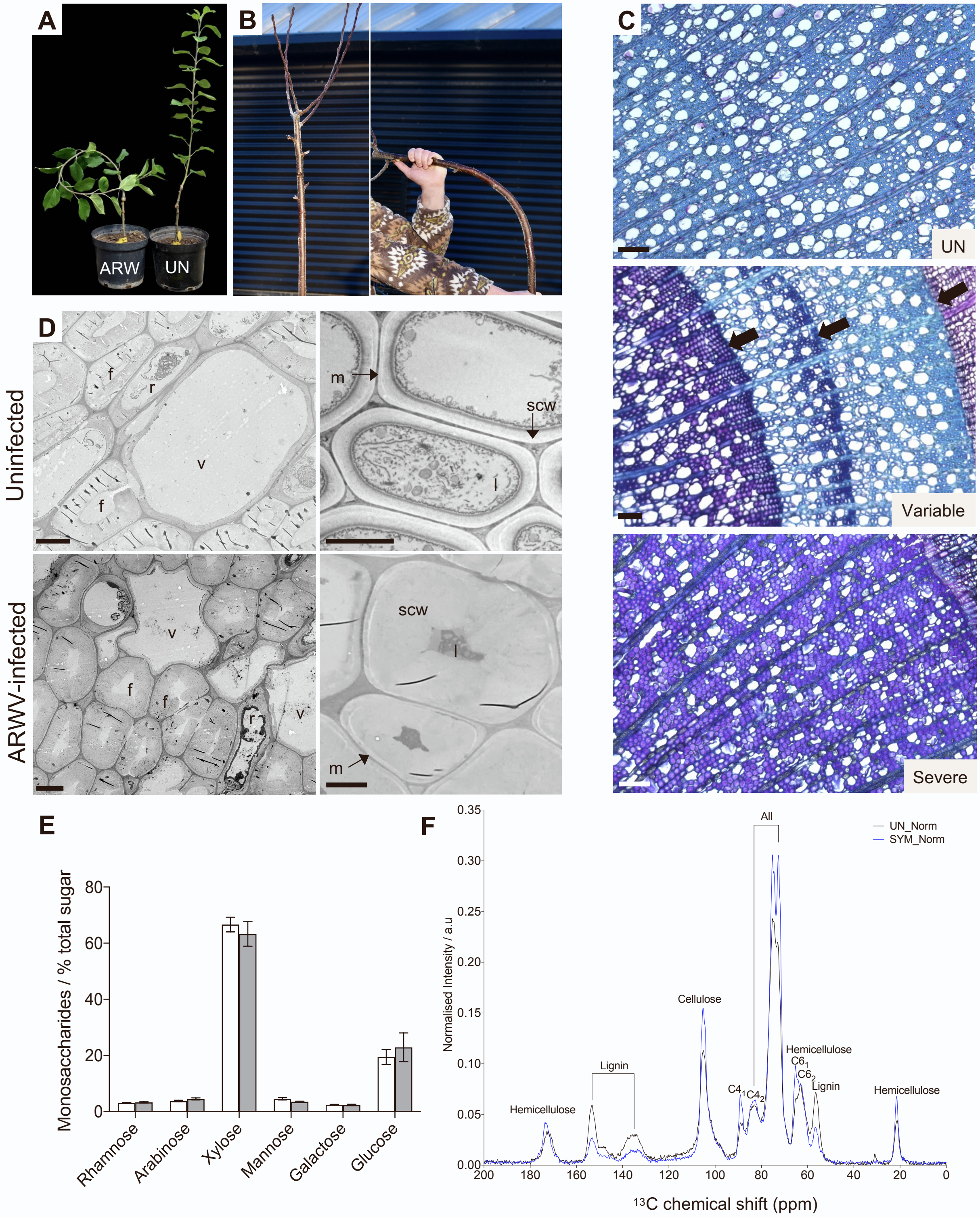

Figure S1 Symptom distribution in ARWV-infected branches and cell wall composition. Related to Figure 1. (**A**) Uninfected (UN) and ARW-infected (ARW) apple tree grafts. The ARW graft has been bent to demonstrate the stem flexibility. **(B)** 4 year old symptomatic branch from ARW-infected apple tree grafts growing unaided (left hand side) and bent to demonstrate the stem flexibility (right hand side) **(C)** Representative apple xylem cross-sections stained with 0.05% Toluidine blue taken from uninfected branches and ARWV-infected branches exhibiting variable or severe symptoms. Asymptomatic regions of infected material resemble the uninfected, while cell walls of symptomatic fibres stain a purple colour. Arrow indicates the growth ring. While year 1 growth exhibits clear symptoms, symptoms are only apparent at the end of year 2 and absent from year 3. Scale bars = 50 µm **(D)** Transmission electron microscopy of apple xylem. Scale bars = 5 µm (left panel) and 3 µm (right panel). Fibres (f), ray cells (r), vessels (v), secondary cell wall (scw), mid lamellae (m) and cell lumen (l) are indicated. **(E)** Monosaccharide composition of xylem from uninfected (white bars) and symptomatic xylem (grey bars). **(F)** Normalized spectra of solid-state ^{13}C NMR analysis from uninfected (black) and symptomatic (blue) cell walls.

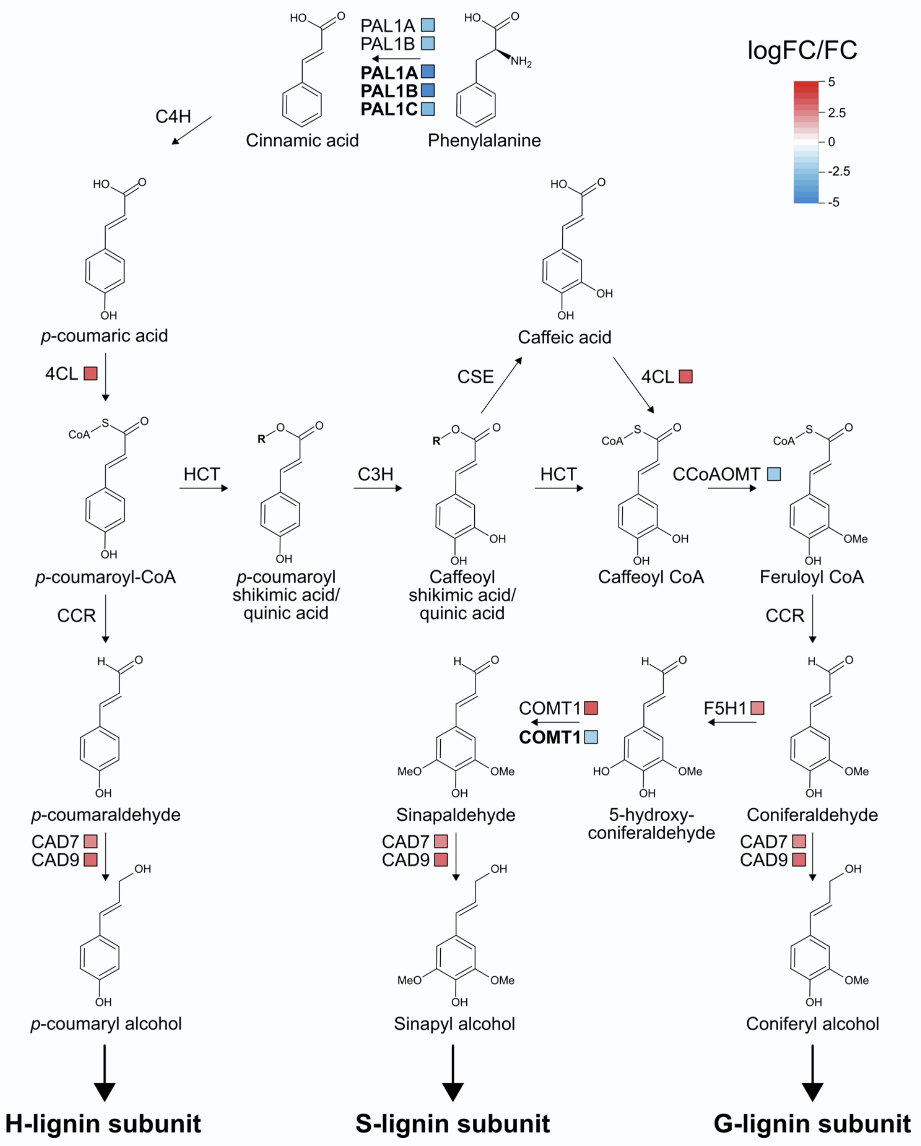

Figure S2 Expression of lignin biosynthetic genes and proteins in apple xylem. Related to Table 1. Each square represents the expression of a different gene (regular font) or protein (bold). Log fold change (FC) in gene expression and FC in protein abundance is expressed in terms of symptomatic xylem relative to uninfected xylem. All genes exhibit significantly altered expression with an FDR-adjusted p-value ≤ 0.01 and logFC ± 2. All proteins exhibit significantly altered abundance with a p-value ≤ 0.05 and FC ± 2.

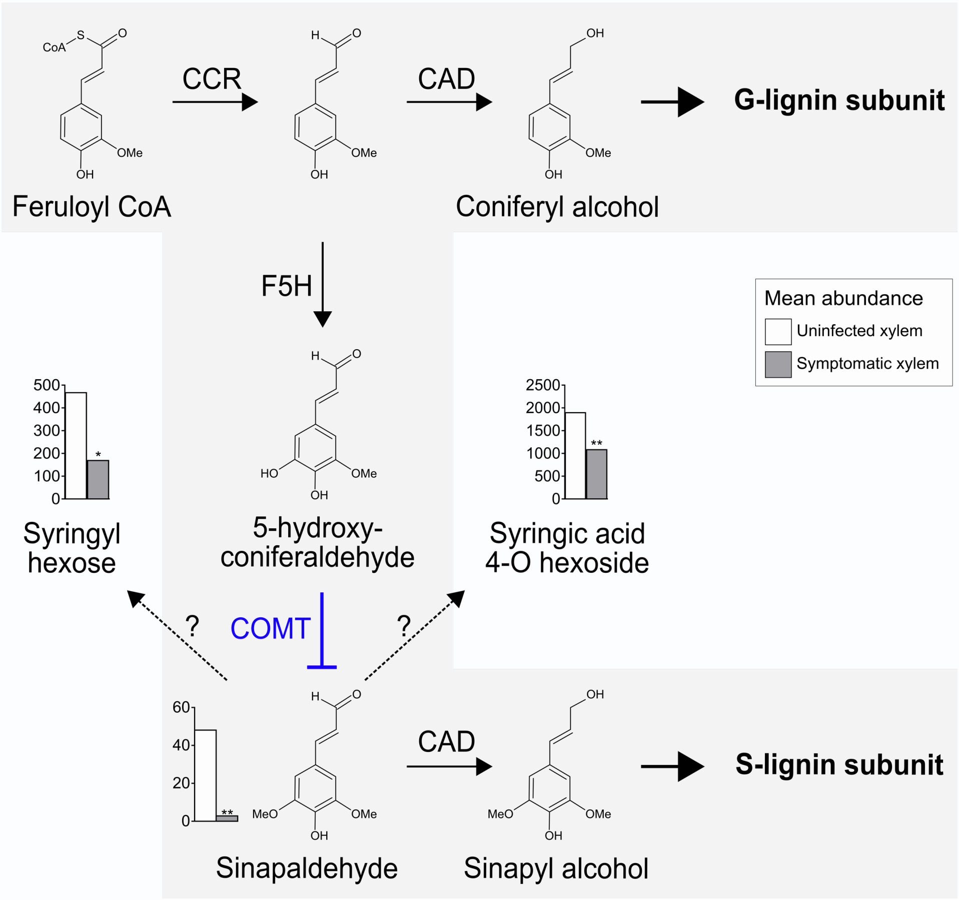

Metabolite ID	Putative name	Type	FC	Mean abundance	
				UN	SYM
2.74_359.0981m/z	syringic acid 4-O-hexoside	P	0.57	1907.56	1093.77
3.52_359.0980m/z	syringoyl hexose	P	0.37	469.20	171.49
9.99_207.0666m/z	sinapaldehyde	P	0.06	48.06	2.83

Figure S3 Disruption in the late stages of lignin biosynthesis in symptomatic xylem. Related to Figure 2. Arrows represent enzymatic steps during the late stages of lignin biosynthesis. Dashed arrows indicate steps that are uncharacterised. Graphs show mean metabolites abundance.. Asterisks indicate significance based on a T-test (*, $p \leq 0.05$, **, $p \leq 0.01$). Table shows metabolites that have a fold-change with a p-value < 0.05. Abbreviations: CCR = cinnamoyl-CoA reductase, CAD = cinnamyl alcohol dehydrogenase, FSH = ferulate 5-hydroxylase and COMT = caffeic acid 3-O-methyltransferase.

Metabolite ID	Putative name	Type	FC	Mean abundance	
				UN	SYM
8.76_687.1558m/z	-	U	17.65	9.12	160.94
8.56_223.0615m/z	2[hydroxy(phenyl)methyl] butanedioic acid	P	11.77	164.88	1941.25
5.12_239.0561m/z	2-[hydroxy(hydroxyphenyl)methyl] butanedioic acid	P	6.62	47.53	314.76
6.48_385.1140m/z	phenylacetyl-hexuronosyl-glycerol	P	5.82	59.07	343.81
7.74_385.1141m/z	phenylacetyl-hexuronosyl-glycerol	P	5.67	365.89	2073.74
15.86_461.1090m/z	-	U	3.91	41.41	161.91
6.36_251.0562m/z	2-oxo-3[hydroxy(phenyl)methyl] pentanedioic acid	P	3.74	137.30	513.18
4.01_465.1032m/z	taxifolin hexoside	F	3.29	120.68	396.67
12.05_883.2292m/z	-	U	2.40	63.23	151.53
1.05_551.1810m/z	-	U	2.39	126.68	302.42
13.94_301.0356m/z	quercetin	F	2.30	44.50	102.37
2.87_359.0617m/z	-	U	2.21	216.96	479.60
3.95_327.1084m/z	dihydro-*p*-coumaric acid hexoside	P	2.20	47.37	103.99
12.05_447.0938m/z	quercetin-O-methyl ether pentoside	F	2.07	156.84	324.62
9.60_793.1869m/z	-	U	2.06	68.22	140.62
11.46_723.1923m/z	-	U	2.04	85.27	173.58
12.97_741.2603m/z	-	U	0.49	238.55	116.02
10.93_615.1853m/z	-	U	0.44	154.62	67.70
7.27_307.1400m/z	-	U	0.41	166.20	67.82
18.44_839.3107m/z	-	U	0.36	193.79	70.44
5.90_419.1559m/z	-	U	0.35	299.06	105.83
16.57_855.3037m/z	tetralignol	O	0.34	230.42	78.22
18.71_809.3003m/z	**G(8-O-4)S(8-8)S(4-O-8)G**	O	0.31	781.92	240.37
16.17_855.3061m/z	**G(8-O-4)S(8-O-4)S(8-5)G**	O	0.29	189.08	55.67
7.64_613.2131m/z	**G(8-O-4)S hexoside**	O	0.27	585.15	157.10
8.24_567.2080m/z	**G(8red-5)G-hexoside**	O	0.25	135.03	34.07
15.69_825.2959m/z	**G(8-O-4)G(8-O-4)S(8-5)G**	O	0.24	366.13	86.07
16.32_825.2952m/z	tetralignol	O	0.22	240.22	52.84

Metabolite ID	Putative name	Type	FC	Mean abundance	
				UN	SYM
2.18_164.0718m/z	phenylalanine	P	18.48	15.71	290.31
7.41_206.0825m/z	N-acetylphenylalanine	P	3.61	20.03	72.39
2.74_359.0981m/z	syringic acid 4-O-hexoside	P	0.57	1907.56	1093.77
9.99_207.0666m/z	sinapaldehyde	P	0.06	48.06	2.83

Table S3 Differentially abundant phenolic metabolites in symptomatic xylem. Related to Figure 2. Fold change (FC) is expressed in terms of symptomatic xylem (SYM) relative to uninfected (UN) xylem. Compounds in bold are annotated with high confidence based on MS/MS spectral library identity matching, and compounds not in bold are putative structures based on MS/MS spectral similarity to library spectra and structural elucidation. The upper table shows metabolites that have a greater than two-fold change in abundance and a minimum abundance of 100 in either symptomatic or uninfected xylem with a p-value ≤ 0.001, while in the lower table the metabolites have a p-value < 0.01. Abbreviations: P = phenylpropanoid, F = flavonoids, O = oligolignols and U = unassigned.

sRNA sequence	ARW-1	ARW-2	ARW-3	ARW-4	Total
CTCTGACTGGTCTCACAACTG	1718	2008	710	170	4606
ATAGTCTACCTGCAGCATTTA	290	616	485	175	1566
CATCACCATCTTTCTAACAAA	260	473	243	89	1065
CCACCGGTTCTTGCATTCT	137	632	196	37	1002
ATAGTCTACCTGCAGCATTTAA	142	359	222	77	800
CTCATGGCTGAAGGAAATTTC	187	386	191	36	800
CTAGAGGGAACATTCGAGAGG	73	360	152	47	632
CCACCGGTTCTTGCATTCTAG	95	387	126	22	630
GCCACCGGTTCTTGCATTCTA	140	192	104	51	487
GCTGTTGGCCACCGGTTCTTG	58	234	141	52	485
CCTTAAGTCATGGAGTCAGCAA	180	168	117	11	476
CATCACAGCTGTTGGCCACCG	111	182	124	21	438
CTAGAGGGAACATTCGAGAGGA	39	273	92	26	430
TCATCACCATCTTTCTAACAAA	70	181	112	63	426
CCTGGACTCTAAGATCAACTT	92	235	50	19	396
ACGACTATAGAAAAACTGCATT	123	151	88	11	373
CCTTAAGTCATGGAGTCAGCA	107	124	91	28	350
GCCACCGGTTCTTGCATTCTAG	67	160	81	34	342
CACCGGTTCTTGCATTCTAGA	61	205	48	18	332
CTTTGCAGAAGAGTCCATTAG	96	155	49	12	312
CATCACCATCTTTCTAACAAAC	49	153	57	50	309
TCATCACCATCTTTCTAACAA	42	133	55	64	294
TCATGCCTAGAGGATCTGAAG	74	121	56	11	262
CAGCTGTTGGCCACCGGTTCT	26	95	85	18	224
TCACTCTGACTGGTCTCACAAC	36	112	46	11	205
TTTCTGCAACTAACTTCAAAGC	47	73	56	18	194
CTGTTGGCCACCGGTTCTTGC	26	85	62	18	191
TAGAGGGAACATTCGAGAGGA	14	110	33	13	170
TCAAGATAAGTAGGCAGCCTTA	31	82	42	11	166
TTGGCCACCGGTTCTTGCATT	22	70	49	25	166
TCACCATCTTTCTAACAAACA	43	67	15	28	153
GAAGTTAAGCCAGCAGATTTA	25	66	33	19	143
GGGAGATTCTGGAACGAGCTA	32	80	19	12	143
CTTAAGTCATGGAGTCAGCAA	27	70	30	14	141
TTCAGCACTGGGGATAATGCC	29	57	40	11	137
ATGCTTTCATCACCATCTTTC	21	45	46	23	135
CATCACCATCTTTCTAACAA	44	35	37	10	126
TTAATCAAGATAAGTAGGCAGC	17	61	28	10	116
GCAATTCAGGTAGTGCAGAGT	32	35	36	12	115
AGCTGTTGGCCACCGGTTCTTG	24	51	19	20	114
TAAGTCATGGAGTCAGCAA	19	61	20	12	112

sRNA sequence	ARW-1	ARW-2	ARW-3	ARW-4	Total
GCAATTCAGGTAGTGCAGAGTG	18	56	18	14	106
TCATCACCATCTTTCTAACA	23	32	19	26	100
TTCATCACCATCTTTCTAACAAA	13	45	18	12	88
TTCATCACCATCTTTCTAACAA	10	38	17	20	85
TTCATCACCATCTTTCTAACA	16	23	29	14	82
TCACCATCTTTCTAACAAAC	21	26	16	10	73
CTGTTGGCCACCGGTTCTTGCA	10	31	15	12	68
TTTCATCACCATCTTTCTAAC	11	15	13	16	55
TAATCAAGATAAGTAGGCAGC	11	21	10	10	52

Table S4 sRNAs mapping to the ARWV. Related to Table 2. All sRNA sequences were found in each ARWV-infected wood sample with more than 10 reads per samples. No reads were detected in UN samples.
